# Chemical Synthesis of the PAX Protein Inhibitor EG1 and Its Ability to Slow the Growth of Human Colorectal Carcinoma Cells

**DOI:** 10.3389/fonc.2021.709540

**Published:** 2021-10-13

**Authors:** Lorissa McDougall, Jui Thiang Brian Kueh, Jake Ward, Joel D. A. Tyndall, Adele G. Woolley, Sunali Mehta, Cherie Stayner, David S. Larsen, Michael R. Eccles

**Affiliations:** ^1^ Department of Pathology, Dunedin School of Medicine, University of Otago, Dunedin, New Zealand; ^2^ Department of Chemistry, University of Otago, Dunedin, New Zealand; ^3^ School of Pharmacy, University of Otago, Dunedin, New Zealand; ^4^ Maurice Wilkins Centre for Molecular Biodiscovery, Auckland, New Zealand

**Keywords:** paired box, PAX, colorectal carcinoma, EG1, treatment, cell cycle, proliferation, apoptosis

## Abstract

Colorectal cancer is primarily a disease of the developed world. The incidence rate has continued to increase over time, reflecting both demographic and lifestyle changes, which have resulted in genomic and epigenomic modifications. Many of the epigenetic modifications occur in genes known to be closely associated with embryonic development and cellular growth. In particular, the paired box (PAX) transcription factors are crucial for correct tissue development during embryogenesis due to their role in regulating genes involved in proliferation and cellular maintenance. In a number of cancers, including colorectal cancer, the PAX transcription factors are aberrantly expressed, driving proliferation and thus increased tumour growth. Here we have synthesized and used a small molecule PAX inhibitor, EG1, to inhibit PAX transcription factors in HCT116 colorectal cell cultures which resulted in reduced proliferation after three days of treatment. These results highlight PAX transcription factors as playing an important role in the proliferation of HCT116 colorectal cancer cells, suggesting there may be a potential therapeutic role for inhibition of PAX in limiting cancer cell growth.

## 1 Introduction

The incidence of colorectal cancer (CRC) in individuals aged 20 to 49 years has continued to escalate throughout Australasia, Europe and Northern America over the last decade (1). It is thought that the ongoing rise in incidence rates may be due to newly evolving lifestyles which correlate with genetic and epigenetic changes (2). The majority of CRC cases worldwide have been recently identified as arising from *de novo* sporadic mutations, as only 5% of individuals have inherited cancer related syndromes and 25% have a family history of CRC (2–4). CRC originates from polyps forming on the mucous membrane of the large intestines which are predisposed to become malignant over time (5). The most common form of CRC is adenocarcinoma, although this disease can also present as other forms of carcinoma, including squamous cell, adenosquamous, spindle cell or undifferentiated carcinoma (2). The polyps can progress into these malignant carcinomas due to the expressional change of genes involved in cell cycling, leading to increased cellular division and proliferation, as well as evasion of cell death (5).

In CRC, as with many other cancers, the genes that often acquire mutations and are associated with the development of tumours include *TP53* (HGNC:11998)*, CTNNB1* (HGNC:2514)*, CDH1* (HGNC:1748)*, TGFB1* (HGNC:11766)*, SMAD4* (HGNC:6770)*, PTEN* (HGNC:9588)*, KRAS* (HGNC:6407) as well as a number of other genes directly related to cell maintenance (4). Additionally, transcription factor genes such as *HNF4A, RAD21* and *PAX2* are dysregulated in CRC, causing changes in the expression of genes associated with tumour growth and metastasis (6, 7). Interestingly, many of these genes and transcription factors are active primarily during embryonic development due to their roles in cellular growth and differentiation (8–12). Therefore, it has been hypothesised that gene activation is often restored in cancers, causing tumours to take on a stem cell-like phenotype. Paired box 2 (*PAX2*) is an example of this, where *PAX2* is normally expressed in embryonic tissues and then subsequently repressed in adults (8, 13). However, in some cancers PAX2 and other PAX proteins are aberrantly overexpressed (8, 14). It has been shown that elevated levels of PAX2 in CRC leads to an increase in the proliferative potential of the cells *via* activation of the transcription factor AP-1 and the JUN-FOS pathway causing the upregulation of cyclin D1, a protein involved in cell cycling (7, 15). Thus, increased proliferation of epithelial cells in the colon can alter the speed at which tumours form due to an increased likelihood that aberrant cell divisions will result in the acquisition of mutations, which then leads to a more progressive cancer (16, 17). The oncogenic properties of PAX can be utilised to develop new cancer treatments by designing therapeutic targets to these proteins in the aim of slowing tumour growth (8, 18–21).

A recent study by Grimley et al. (2017) discovered a small compound (EG1) with the ability to inhibit the DNA binding and hence transcriptional activity of PAX proteins, resulting in arrested growth of the embryonic ureteric bud in a mouse developmental explant model. While EG1 was shown to inhibit Pax2, it is possible that EG1 also targets other Pax proteins (e.g. Pax5, Pax8, and possibly Pax6), due to high sequence homology between the Pax isoforms. Additionally, the structure of EG1 was modelled using the DNA binding site of an *in silico* PAX2 model designed from the PAX5 paired domain (22). Therefore, as EG1 is able to inhibit Pax transcription factors, we hypothesized that EG1 could reduce human colorectal cancer cell proliferation due to it having a binding affinity (K_d_) of 1.5 μM to the PAX2/PAX5/PAX8 protein DNA binding domain *via* steady state analysis (22). EG1 would thus interfere with the PAX protein’s ability to interact with DNA causing failure of polymerase recruitment, and consequently resulting in a loss of target gene transcription (22). When tested *in vitro* EG1 was shown to have an IC_50_ of ~10 μM and cause changes in the expression of several known Pax2 regulated genes, as well as a decrease in phosphorylated histone 3, an indicator of proliferation (22). Given the features described, we therefore tested our hypothesis to investigate whether EG1 would inhibit the proliferation of *in vitro* cultured HCT116 colorectal carcinoma cells. Ultimately, by slowing cell proliferation EG1 could theoretically reduce the growth of tumours, and subsequently improve the overall survival rates of patients. Furthermore, targeting PAX proteins may cause fewer side effects in normal adult tissues which have less requirement of *PAX* gene expression than fetal tissues, thus providing better treatment outcome.

We show here that EG1 treatment was able to successfully slow HCT116 colorectal carcinoma cell proliferation, and we present evidence that this occurs by specifically targeting PAX proteins involved in regulating cell proliferation.

## 2 Methods

### 2.1 EG1 Synthesis

#### 2.1.1 Methyl 2-(4'-nitrobenzamido)benzoate (3)

**Figure 1:**
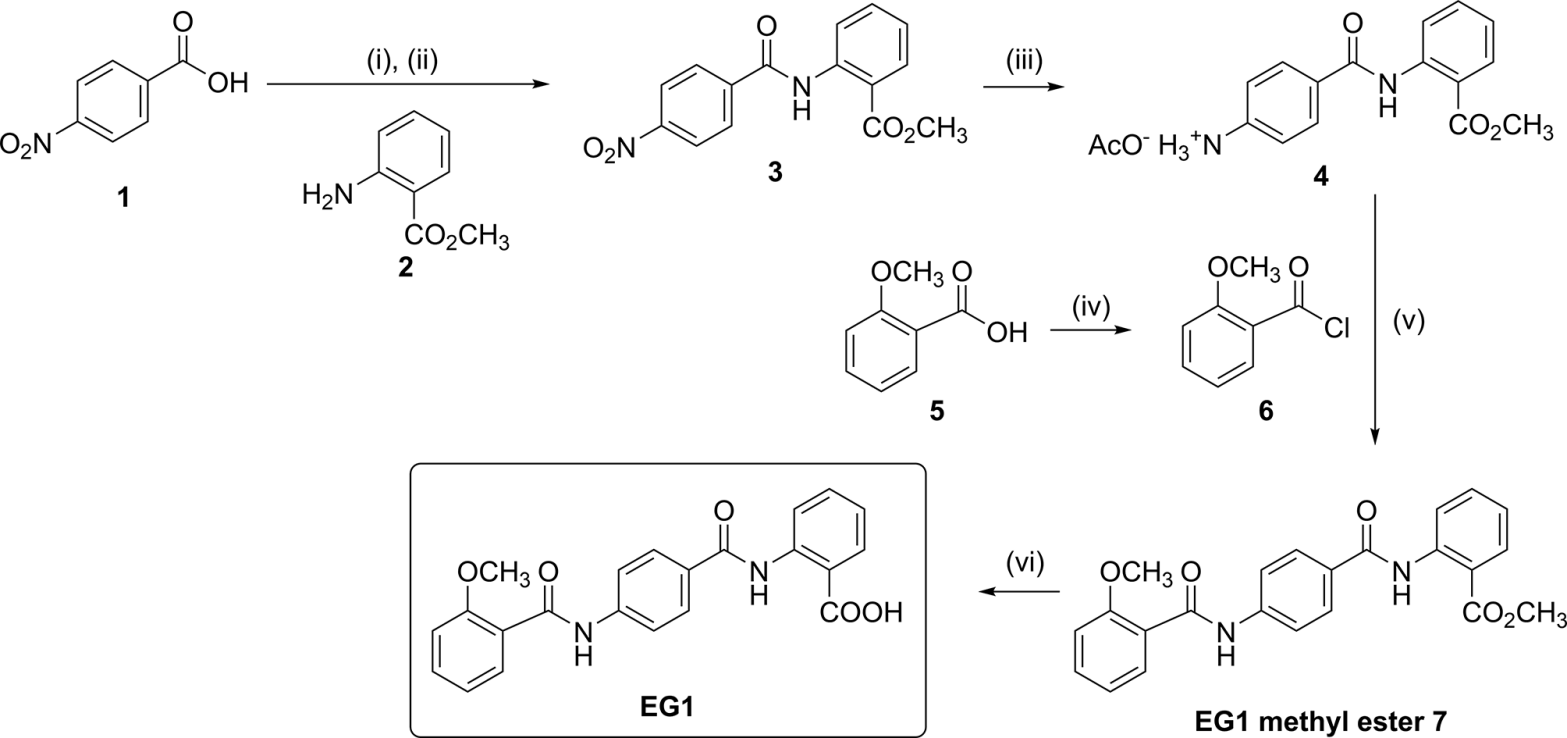
Chemical synthesis of EG1. Reagents and conditions: (i) SOCl_2_, DMF, CH_2_Cl_2_, reflux, 6 h; (ii) 2, DIPEA, CH_2_Cl_2_, 0°C to rt, 5 h, 68%; (iii) H_2_, Pd/C (10% wt.), AcOH:CH_2_Cl_2_:EtOAc (1:10:30), 35-40°C, 22 h, 79%; (iv) (COCl)_2_, DMF, CH_2_Cl_2_, reflux, 1.5 h; (v) DIPEA, DMF:CH_2_Cl_2_ (1:4), 0°C to rt, 69 h, 60%; (vi) 0.5 M aqueous NaOH, THF, 30°C to rt, 16 h, 70%.

Thionyl chloride (940 μL, 11.99 mmol) was added to a suspension of 4-nitrobenzoic acid (**1**) (1 g, 5.98 mmol) in anhydrous CH_2_Cl_2_ (30 mL) at room temperature (rt). A catalytic quantity of DMF (two drops) was added to the reaction mixture at rt, effervescence resulted, and the reaction mixture was heated to reflux. After six hours the reaction mixture had dissolved to a clear solution which was allowed to cool to rt and concentrated *in vacuo* to afford the acid chloride as a yellow solid. Without further purification, the acid chloride was then immediately dissolved in anhydrous CH_2_Cl_2_ (25 mL). A solution of methyl 2-aminobenzoate (**2**) (1.07 g, 7.06 mmol) and anhydrous DIPEA (2.2 mL) in anhydrous CH_2_Cl_2_ (20 mL) was cooled to 0°C and the acid chloride solution was added over five minutes. The reaction mixture was then heated to reflux for five hours and then cooled to 0°C. 1 M aqueous HCl (50 mL) was slowly added, and the aqueous phase was extracted with CH_2_Cl_2_ (3 x 50 mL). The combined organic extracts were washed sequentially with 1 M aqueous HCl (50 mL) and H_2_O (2 x 50 mL), then dried over Na_2_SO_4_ and concentrated *in vacuo* to obtain a yellow solid. Purification by crystallisation from a mixture of hot CH_2_Cl_2_ (30 mL) and petroleum ether (20 mL) gave yellow crystals which were isolated by Büchner filtration to afford title compound **3** (1.22 g, 68%) (**Figure 1** and **Supplementary Figure 11.4**).

#### 2.1.2 4'-((2-(Methoxycarbonyl)phenyl)carbamoyl)benzenaminium Acetate (4)

Palladium on charcoal (10 % weight, 177 mg, 0.17 mmol) was added to a suspension of **3** (0.5 g, 1.67 mmol) in a mixture of AcOH:CH_2_Cl_2_:EtOAc (1:10:30, 41 mL) and flushed with N_2_ gas for 10 minutes. H_2_ gas (1 atm) was then introduced, and the reaction mixture heated to 35–40°C. After 22 hours the reaction mixture was flushed with N_2_ gas for 10 minutes and the mixture was filtered through Celite. The Celite was washed with CH_2_Cl_2_ (75 mL), and the organic filtrate concentrated *in vacuo* to obtain *title compound* **4** as a white solid (432 mg, 79%)(**Figure 1** and **Supplementary Figure 11.5**).

#### 2.1.3 Methyl 2-(4'-(2''-methoxybenzamido)benzamido)benzoate (EG1 Methyl Ester 7)

**Figure 2:**
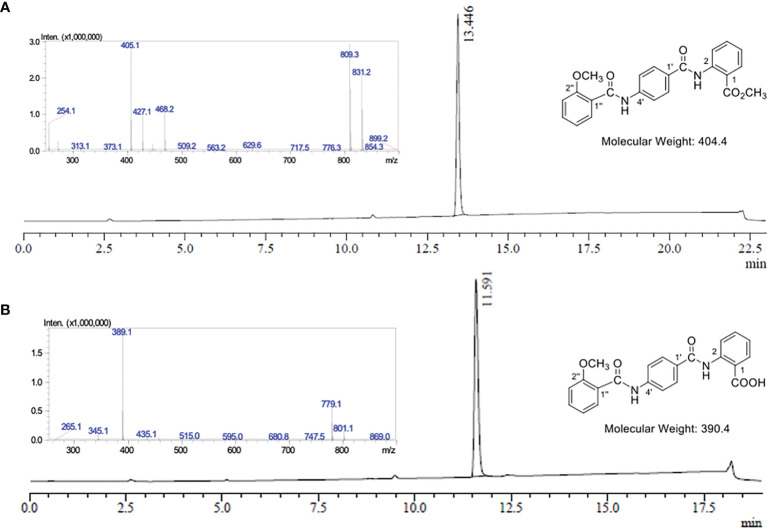
LC-MS analysis of EG1 methyl ester 7 and EG1. **(A)** UV chromatogram from the LC-MS of EG1 methyl ester 7 at λ = 254 nm. *LC-MS conditions*: Phenomenex Prodigy column (C–18, 5 μm, 3.00 × 250 mm) at 0.5 mL/min and heated to 40°C. 10% to 100% B over 12.5 min, then 100% B for 6.5 min, 100% to 10% B over 1 min, then 10% B for 3 min. The solvent system for LC purposes was a mixture of A (0.1% formic acid in H_2_O) and B (0.1% formic acid in CH_3_CN). *Insert:* Mass spectrum in positive ionisation mode at 13.45 mins; MS *m/z* (ESI+): 405 ([M + H]^+^, 100%), 427 ([M + Na]^+^, 34), 809 ([2M + H]^+^, 93), 831 ([2M + Na]^+^, 72). **(B)** UV chromatogram from the LC-MS of EG1 at λ = 254 nm. *LC-MS conditions*: Phenomenex Prodigy column (C–18, 5 μm, 3.00 × 250 mm) at 0.5 mL/min and heated to 40°C. 10% to 100% B over 12.5 min, then 100% B for 2.5 min, 100% to 10% B over 1 min, then 10% B for 3 min. The solvent system for LC purposes was a mixture of A (0.1% formic acid in H_2_O) and B (0.1% formic acid in CH_3_CN). *Insert*: Mass spectrum in negative ionisation mode at 11.59 mins; MS *m/z* (ESI–): 389 ([M – H]^-^, 100%), 779 ([2M – H]^-^, 28).

Oxalyl chloride (190 μL, 2.27 mmol) was added to a solution of 2-methoxybenzoic acid (**Figure 1.5**) (240 mg, 1.54 mmol) in anhydrous CH_2_Cl_2_ (8 mL) at rt. A catalytic quantity of DMF (two drops) was added to the reaction mixture at rt to result in effervescence. The reaction mixture was stirred at rt for 1.5 hours and then concentrated *in vacuo* to afford acid chloride (**Figure 1.6**) as a yellow oil. Without further purification, acid chloride was immediately diluted in anhydrous CH_2_Cl_2_ (7 mL) and cooled to 0°C. In a separate round bottom flask, a solution of amine acetate salt **4** (503 mg, 1.52 mmol) was prepared by the subsequent addition of anhydrous DMF (3 mL), DIPEA (620 μL, 3.56 mmol) and then anhydrous CH_2_Cl_2_ (12 mL). The resulting solution of amine acetate **4** was then added slowly over five minutes to the cooled solution of acid chloride **6**. The reaction mixture was then allowed to warm to rt and stirred for 69 hours and then concentrated *in vacuo*. The residue was taken up in saturated NH_4_Cl (20 mL) and the aqueous phase extracted with CH_2_Cl_2_ (3 x 50 mL). The combined organic extracts were washed with H_2_O (80 mL), then dried over Na_2_SO_4_ and concentrated *in vacuo* to obtain a yellow oil. Purification was performed first with flash chromatography (CH_2_Cl_2_ load, 0%, then 35%, then 40% EtOAc in petroleum ether, then 50% EtOAc in CH_2_Cl_2_) (**Supplementary Figure 11.6**). The fractions containing the product were combined and concentrated *in vacuo* to afford a yellow solid. This was purified further by trituration from CH_2_Cl_2_ (20 mL) and petroleum ether (40 mL) to yield EG1 methyl ester **7** as a pale orange-white powder (358 mg, 60%) (**Figure 1** and **Figure 2A**).

#### 2.1.4 2-(4'-(2''-Methoxybenzamido)benzamido)benzoic Acid (EG1)

0.5 M aqueous NaOH (1.5 mL, 0.74 mmol) was added to a suspension of EG1 methyl ester (**7**) (100 mg, 0.25 mmol) in THF (5 mL) at rt. The reaction mixture was stirred at 30 ⁰C for 10 minutes to dissolve all solids and then allowed to stir at rt. After 16 hours, the reaction mixture was acidified with 1 M aqueous HCl to pH 1-2 and extracted with EtOAc (3 x 50 mL). The combined organic extracts were washed with H_2_O (50 mL), then saturated NaCl (50 mL) and then dried over MgSO_4_. The volume of the organic extracts was then reduced *in vacuo* to approximately 5-10 mL (but not to dryness) to obtain a white precipitate which was isolated by Büchner filtration. The precipitate was washed with EtOAc (3 x 10 mL) and dried to afford EG1 as a white powder (68 mg, 70%) (**Figure 1**, **Figure 2B** and **Supplementary Material 11.7**).

### 2.2 LC-MS Selective Ion Monitoring

Mass spectrometry grade acetonitrile (CH_3_CN) and water (H_2_O) was purchased from ThermoFisher Scientific (MA, USA) and mass spectrometry grade formic acid was purchased from Merck (Darmstadt, Germany). RP-LC-MS analyses were conducted on an analytical RP-HPLC (Shimadzu LC–20AD equipped with a SPD-20A UV detector [210 and 254 nm] and a Shimadzu LC-MS-2020 Liquid Chromatograph Mass Spectrometer operating in negative ionisation mode with Selective Ion Monitoring [SIM] for the *m/z* values of 389 and 779) using a Phenomenex Prodigy column (C–18, 5 μm, 3.00 × 250 mm) at 0.5 mL/min and heated to 40°C. The solvent system for LC purposes was a mixture of 0.1% formic acid in H_2_O and 0.1% formic acid in CH_3_CN. The LC method used to analyse the EG1 standards and samples: 10% to 100% B over 12.5 min, then 100% B for 2.5 min, 100% to 10% B over 1 min, then 10% B for 3 min unless stated otherwise. The total ion current (TIC) generated from the sum of SIM *m/z* values of 389 and 779 at 11.6 minutes was then integrated for analysis (see section 2.8).

### 2.3 Selective Ion Monitoring of EG1 in Cell Culture

The HCT116 cells were grown on 6-well Grenier plates for a total of four days with EG1 or solvent control (DMSO) treatment every 24 hours (see section 3.4). On the third day of treatment samples from each media condition were taken for analysis of pre-treatment concentration and the remaining solutions were used to treat the cells. After a subsequent 24 hours the media was removed from the EG1 treated and DMSO control cultures as the post-treatment media samples, and the cytoplasmic and nuclear lysates were extracted from the cells. Prior to cell lysis the EG1 and control cell cultures were harvested using 0.25% Trypsin-EDTA and centrifuged for 5 minutes at 350rpm to produce a pellet which was further washed with PBS (pH 7.4). The cell pellet was then resuspended in RIPA buffer and subjected to constant agitation on an orbital shaker in room temperature for 30 minutes to lyse the cell membrane. The samples were then briefly vortexed (5 seconds) and placed in a water bath sonicator for three minutes at 35°C. The samples were removed and vortexed again (5 seconds) to resuspend cellular material and then sonicated for another four minutes at 35°C. To retrieve the supernatant the samples were centrifuged at 12,000 rpm for 20 minutes at 4°C, transferred to a new tube and kept at 4°C until measurement by SIM.

Each sample to be analysed was then filtered through a 0.45 µm nylon syringe filter and 2 µL was injected for LC-MS analysis using SIM. The total ion current (TIC) generated from the sum of the SIM *m/z* values 389 and 779 at 11.6 minutes was integrated to provide a ’y’ value (see section 2.8). The value for y was then inserted into the equation x = y/175573 to give an approximate concentration ‘x’ of EG1(**Supplementary Figures 12.4, 12.5**).

### 2.4 Cell Culture

This study uses the human colorectal carcinoma cell line HCT116 originally obtained from the American Tissue Culture Collection (ATCC, Manassas, VA, USA) and recently verified by DNA Diagnostics as having an 89% match to the reference (**Supplementary Figure 12.6**), as well as testing negative for Mycoplasma by metabolite and PCR based assays. Initially we utilised an HCT116-fluorescent, ubiquitination-based cell cycle indicator (FUCCI) cell line that was created elsewhere (23, 24). However, this feature was not used for the cell cycling experiments and had no impact on the fluorescent detection of apoptosis, proliferation or cell viability staining during FACS collection using a Beckman Coulter Gallios cytometer, as the mCherry and mVenus fluorophores were unable to be detected by the lasers (**Supplementary Figure 12.9**). The HCT116 cells were grown on plastic with filtered Dulbecco’s modified eagle media (DMEM, ThermoFisher, MA, USA) supplemented with 1 x non-essential amino acids (ThermoFisher, MA, USA), 1 x Gluta-Max (ThermoFisher, MA, USA) and 10% fetal bovine serum (FBS, Moregate Biotech, Hamilton, NZ). Prior to treatment the cells were grown for five passages and then plated at staggered starting densities to ensure they remained in the growth phase over the four days. To do this; 500,000 cells were plated for day 0, 250,000 cells were plated for day 1, 125,000 cells were plated for day 2, 75,000 cells were plated for day 3, and 37,500 cells were plated for day 4. The cultures were then exposed to 25 μmol/L EG1 (treated) or an equal volume of DMSO (solvent control), as previously determined by Grimley et al. (2017), with treatments every 24 hours for a total of 96 hours. The HCT116 cells were also treated with EG1 at 250 μmol/L, 10-fold that of the original concentration, to show an exaggerated effect of what was observed with the lower 25 μmol/L EG1. Also, to identify phenotypic changes the 250 μmol/L EG1 treated cells were imaged by phase contrast using the Lionheart FX microscope (BioTek, VT, USA).

### 2.5 RNA Extraction, cDNA Conversion and Real Time Quantitative PCR

Real time quantitative PCR (RT-qPCR) was used in this study to determine *PAX* expression in the HCT116 cell line, with comparison to a melanoma cell line (UACC62) and a prostate cancer cell line (PC-3) (**Supplementary Figure 12.7**). Prior to RT-qPCR, RNA was extracted from the cultures using an RNeasy mini kit (QIAGEN, MD, USA) as per the product instructions, and measured for concentration and purity on a Nanophotometer (Implen, Munich, Germany). The RNA was further diluted to 10 ng/µl for conversion into cDNA following the reverse transcription high-capacity cDNA kit (ThermoFisher, MA, USA) protocol, and used for RT-qPCR. The RT-qPCR assay included primers targeting three housekeeping genes, Hypoxanthine Phosphoribosyltransferase 1 (*HPRT1*), Beat-2-Microglobulin (*B2M*) and Ubiquitin C (*UBC*), as well primers targeting the Paired box genes (*PAX2*, *PAX5*, *PAX6* and *PAX8*)(**Supplementary Table 12.8**). Additionally, expression of the likely downstream PAX targets Cadherin 2 (*CDH2)*, Tumour Protein 53 (*TP53)* and Transforming Growth Factor-β Receptor 1 (*TGF-βR1)* were identified by RT-qPCR (**Supplementary Table 12.8**). The primers for each gene were used at a final concentration of 0.2 μmol/L in nuclease free water and TB SYBR green (Medi’Ray, Auckland, NZ). The RT-qPCR itself was carried out using the Roche LightCycler 480 and analysed using qbase+ software, version 3.0 (Biogazelle, Zwijnaarde, Balgium).

### 2.6 Proliferation and Apoptosis Assay

Proliferation and apoptosis in the HCT116 cells were identified using fluorescent staining and flow cytometry (**Supplementary Figure 12.9**). To determine proliferation in the HCT116 cells CellTrace violet (ThermoFisher, MA, USA) was used to stain the membrane and measure dye dilution as a result of cellular division. To identify apoptosis in the cultures Annexin-V APC (ThermoFisher, MA,USA) was used to stain phosphatidylserine, a membrane bound apoptosis marker. Additionally, the cells were stained with propidium iodide (PI) (BioLegend, CA, USA) to allow for the isolation of living cells to be used in proliferation analysis, and to determine the apoptotic cells *via* a PI *versus* Annexin scatter plot. On day zero the previously plated HCT116 cells were stained with CellTrace and given their first treatment of EG1 or solvent control (DMSO). Following this the cells were harvested or treated every 24 hours for a total of 96 hours (see section 3.4), and those harvested were stained with the cell viability marker propidium iodide (BioLegend, CA, USA) as well as Annexin-V APC, and then resuspended in annexin binding buffer. 50,000 stained cells were further processed through the Beckman Coulter Gallios flow cytometer and the data was analysed using Kaluza analysis software (version 2.1.1, 2018) and Rstudio (version 1.4, 2020), with division rate distribution being calculated *via* the precursor cohort model (25) (section 2.8, **Supplementary Figure 12.10**).

### 2.7 Cell Cycling Assay

Cell cycling in HCT116 cells was measured *via* propidium iodide (PI) staining intensity and flow cytometry for the detection of changes in DNA amount per cell to indicate cell cycle phase (26). The HCT116 cells were plated for each time point at staggered densities to ensure that similar cell numbers were retrieved every 24 hours and that the cells remained dividing over the full length of the assay. After 24 hours of growth the cells were blocked at the G1 phase by 2mmol/L thymidine treatment for 16 hours and then released with working media for 9 hours to allow the cells to continue cycling. Once the cells had been released, they were again treated with 2mmol/L thymidine for 16 hours to block any cells that may have escaped G1 at first, and then immediately treated with DMSO (control) or EG1 (treatment) to begin the cell cycling assay. At each time point (0 hours, 24 hours, 48 hours, 72 hours and 96 hours) the cells were harvested and stained with zombie yellow viability dye (BioLegend, CA, USA) as per the product instructions, followed by fixation for 30 minutes with 70% ethanol and then PI staining in FACS buffer prior to collection of the cells using a Beckman Coulter Gallios flow cytometer. During collection the zombie yellow negative living cells were isolated to prevent false positives, and the voltage for PI staining was determined by the G0/G1 phase peak assuming that these cells had single chromosomal copies and so were considered to have the lowest PI fluorescence. Alternatively, cells in the G2/M phases have twice as many chromosomes during duplication and cell division and so produce a fluorescent PI peak at the highest intensity. Thus, the cells that have a staining intensity between these two phases were considered to be in the middle of duplicating and therefore are in the S phase. Gates for each phase were set using Kaluza analysis software (version 2.1.1, 2018) to calculate the percentage of cells in each phase to then be plotted using Rstudio software (version 1.4, 2020) (see section 2.8).

### 2.8 Statistical Analyses

#### 2.8.1 Mass Spectrometry Selective Ion Monitoring Analysis

LC-MS Selective Ion Monitoring for quantification of the EG1 compound *in vitro* was measured by producing a standard curve of stock and standard EG1 solutions. To do so, EG1 (1.96 mg) was first dissolved in DMSO (0.5 mL) and CH_3_CN (4.5 mL) to create the stock solution of EG1 (1 mmol/L). The stock solution was diluted 1 in 10 using CH_3_CN to give a standard solution of EG1 (100 µmol/L), this was then used to prepare the other standard solutions by serial dilution using CH_3_CN (50, 25, 12.5, 6.25, 3.13 1.56, 0.78, 0.39 µmol/L). Each standard solution was filtered (0.45 µm nylon syringe filter) and then 2 µL was injected for LC-MS analysis using SIM. The total ion current (TIC) generated from the sum of the SIM *m/z* values 389 and 779 at 11.6 minutes was integrated and used to generate the standard curve (**Supplementary Figure 12.2**) that is described by equation (1).


(1) $$\begin{array}{l} \text{y}=175573\;x\;\text{where y}=\text{peak area integrated TIC at }11.6\;\text{minutes}\\ \text{x}=\text{concentration of EG}1\text{ (}µ\text{mol}/\text{L}) \end{array}$$

Equation (1) is rearranged to give equation (2) which provides an approximate concentration of EG1 (x).


(2) $$\text{x}=\frac{\text{y}}{175573}$$

#### 2.8.2 Apoptosis Analysis

Apoptosis in the HCT116 cells with control (DMSO) or EG1 treatment was measured *via* flow cytometry by gating on the cells with high Annexin V APC fluorescence. This was done using 24 hour 25 μmol/L Doxorubicin treated control cells as a positive control for apoptosis which set a baseline for Annexin expression and allowed us to gate on positive cells in the EG1 treated and controls. Using Kaluza analysis software (version 2.1.1, 2018) we were able to calculate the percentage of cells that fall within the apoptosis positive gate to then plot control *versus* EG1 for each time point in Rstudio (version 1.4, 2020) using the raw percentage values.

#### 2.8.3 Proliferation Analysis

To measure proliferation in the EG1 treated and control HCT116 cell cultures a histogram of cell number *versus* CellTrace violet intensity was produced during FACS collection. The control histograms from each time point over a total of 96 hours were used to set the boundaries of each gate either side of the peak from which the number of cells were measured within each division (0 to 5) per collection event (**Supplementary Figure 12.10**). These values were then used to calculate the distribution of cell division in the populations using the precursor cohort basic method (25) which introduces a continuous scale to adjust for the likelihood that cells are between divisions (i.e 1.5 rather than 1). To calculate the proportion of cells in each division the raw values from each gate (x) were used in the equation x/2*^i^* where *i* is the division number. The adjusted values were then shown as a histogram with the cell number (proportion) against the divisions for each time point for both the controls and the EG1 treated.

### 2.9 Cell Cycling Analysis

Cell cycling in the HCT116 cells was measured *via* PI staining of DNA whereby the dividing cells (G2/M) contain double the amount of DNA as those in the growth phases (G0/G1), and therefore are detected by flow cytometry at differing fluorescent intensities. During collection of the PI and zombie yellow (BioLegend, CA, USA) stained cells the living cells (zombie negative) were gated on to remove any false positives in the PI cell cycling plots. Two cell cycling plots were used to detect the shift in population fluorescence, the first being a scatter plot of PI *versus* forward scatter area to visualise the density and spread of PI-stained cells, and the second being a histogram of cell count *versus* PI to measure the percent of cells in each phase (**Supplementary Figure 12.2**). The histogram was then used to analyse the change of cell percentages per cell cycle phase (G0/G1, S or G2/M) in the EG1 treated compared to the control populations. This analysis was done using Kaluza analysis software (version 2.1.1, 2018) by setting gates that encompass the peaks which appear for the G0/G1 phases and S phase; however a defined G2/M phase occurred only at 96 hours. The analysis software was then able to measure the percentage of cells within each of these gates to produce the values which were used to make the cell cycling figure in Rstudio (version 1.4, 2020).

## 3 Results

### 3.1 Synthesis of the EG1 Compound Verified by Mass Spectrometry

No preparation details for EG1 have been published to date, thus synthesis was completed in-house from readily available starting materials. Firstly, 4*-*nitrobenzoic acid (**1**) was treated with thionyl chloride, and the resulting acid chloride was reacted with methyl 2-aminobenzoate (**2**) to give the nitro-adduct **3** (**Figure 1**).

Reduction of the nitro group using hydrogen in the presence of palladium on carbon gave the amine **4** as its acetate salt. To attach the final aromatic ring needed for EG1,

2-methoxybenzoic acid (**5**) was converted into acid chloride **6** which was immediately reacted with the amine acetate **4** to yield the EG1 methyl ester **7** (**Figures 1**, **2**). Finally, hydrolysis of the methyl ester under basic conditions afforded EG1 (see **Supplementary Methods** for experimental and characterisation data).

**Figure 3:**
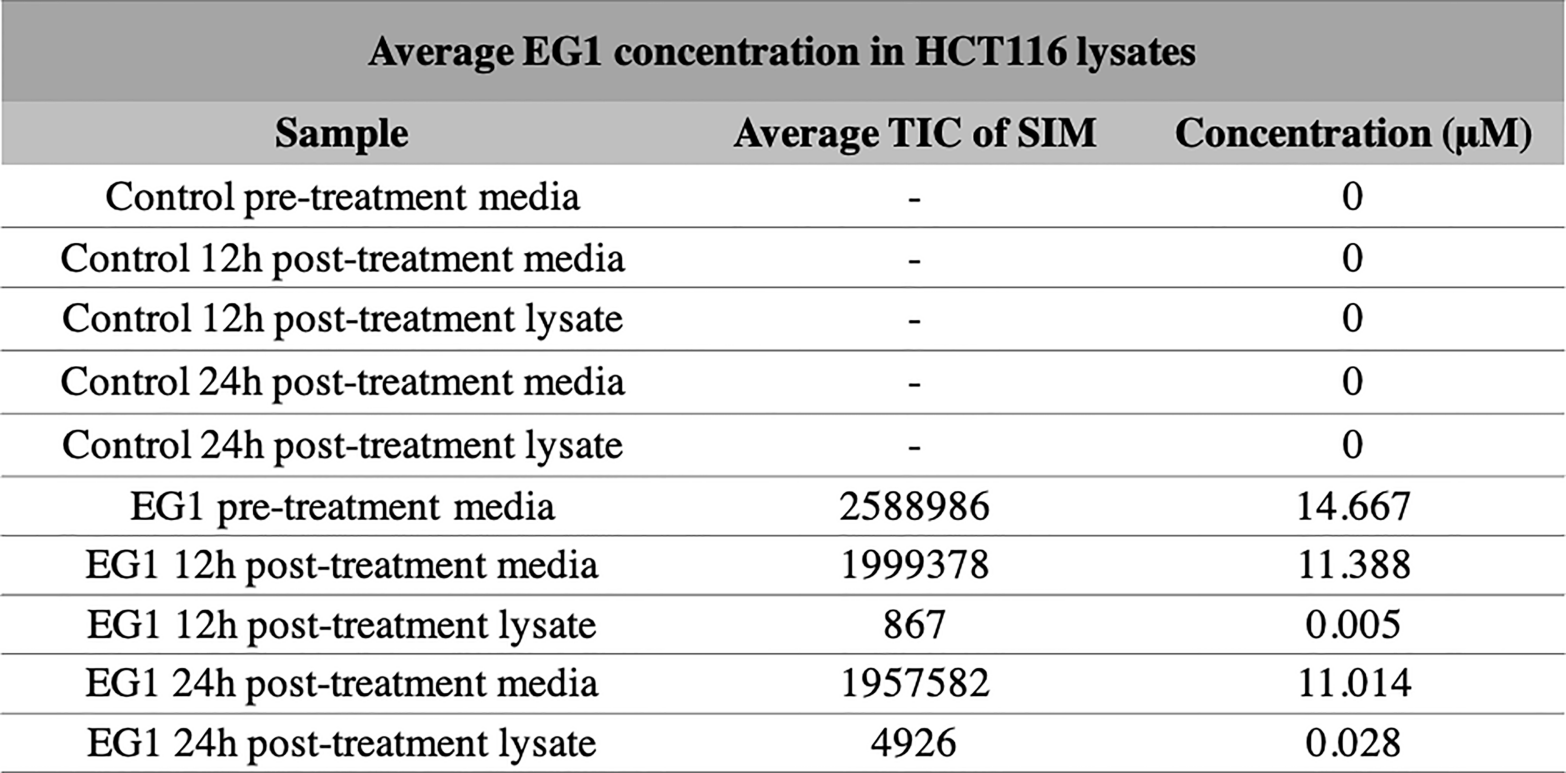
Selective ion monitoring mass spectrometry of EG1 in media and cell lysates post-treatment. The concentration of EG1 in the treatment medias and cell lysates of HCT116 cells were measured using selective ion monitoring mass spectrometry to determine EG1’s stability in media and it’s uptake into cells. EG1 was measured in both the control and EG1 drug conditioned pre-treatment medias as well as in post-treatment medias recovered from cell cultures after 12 hour and 24-hour incubations. There was no detection of EG1 in the control medias throughout the incubational periods. In the drug conditioned pre-treatment medias the average concentration of EG1 was ~10 μM lower than the expected 25 μM (n=2). Additionally the post-treatment medias at both 12 hours and 24 hours had reduced by 3μM from the pre-treatment concentration yet we observed EG1 uptake of only 0.005 μM and 0.028 μM respectively into the HCT116 cells (n=4).

Selective ion monitoring mass spectrometry was used to identify a stable EG1 product in the cell culture medium after 12 hours and 24 hours, as well as successful uptake of the compound into HCT116 cell culture. In the initial media sample taken immediately after the addition of EG1 to a concentration of 25 mol/L, we measured a lower true EG1 concentration of 14.7 μmol/L on average (**Figure 3**). Additionally, we measured the EG1 content of this same media after culturing with the HCT116 cells for both 12 hours and 24 hours and were able to see an average reduction to 11.4 μmol/L and 11 μmol/L respectively (**Figure 3**). We also measured detectable EG1 in the HCT116 cell lysate and found that 0.005 μmol/L of EG1 had entered the cells at 12 hours and 0.028 μmol/L at 24 hours (**Figure 3**).

### 3.2 The Human Colorectal Cancer Cell Line HCT116 Expresses *PAX* Genes

**Figure 4:**
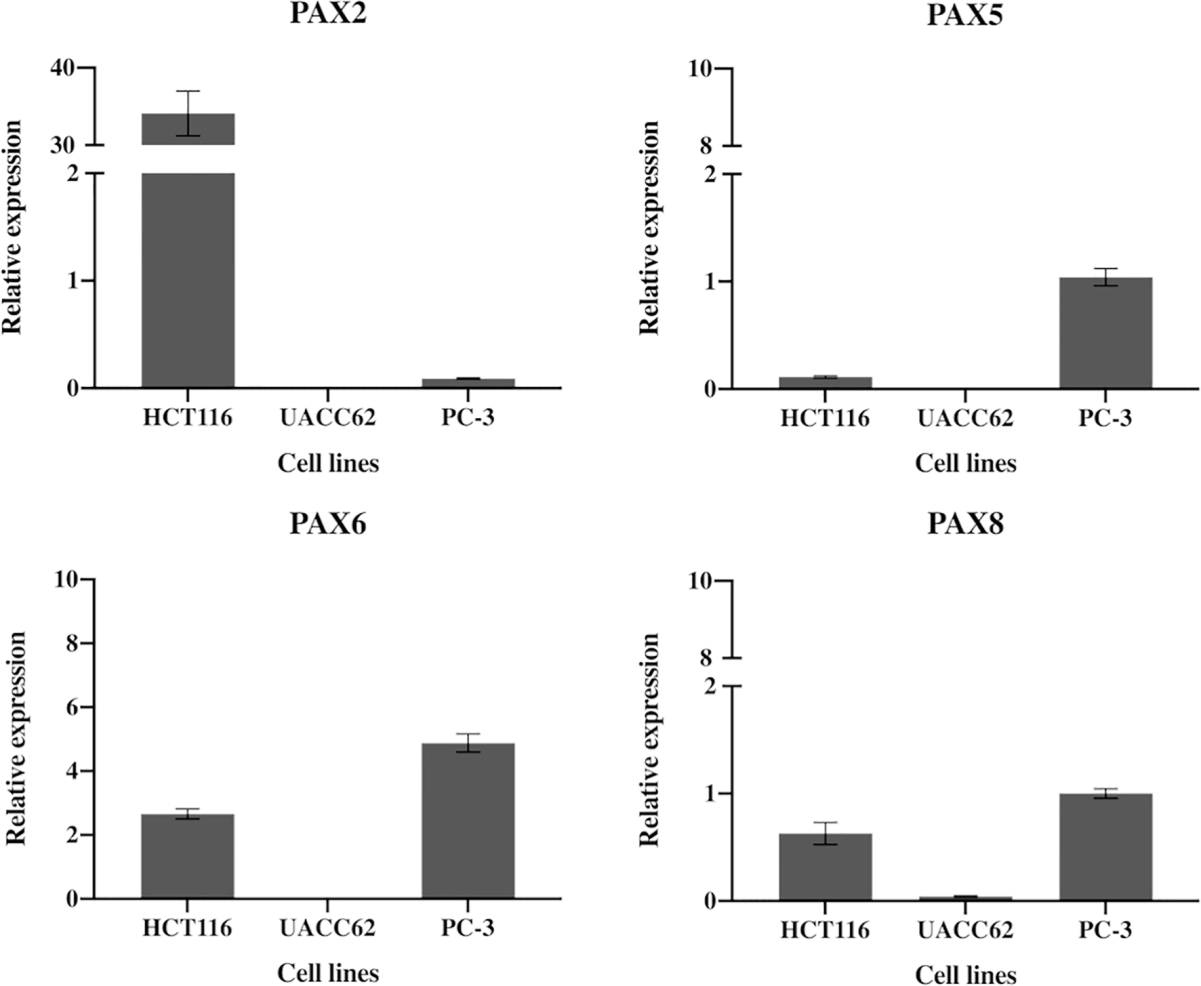
Expression of *PAX2, PAX5, PAX5* and *PAX8* genes in HCT116 cells. HCT116 and two additional cell lines (MCF10A and PC-3) show differential expression of the PAX genes likely to be inhibited by EG1. The HCT116 cells express PAX2 34-fold and PAX6 2.7-fold more than the housekeepers (B2M, HPRT1 and UBC). HCT116 also express both PAX5 (0.1) and PAX8 (0.6) although both were detected at very low levels (n=3).

We used RT-qPCR to determine the relative expression levels of four paired box (*PAX*) genes which are most likely to encode binding targets for EG1 in colorectal carcinoma cells (*PAX2, PAX5, PAX6, PAX8*). This analysis showed that the human cell line HCT116 expressed *PAX2* at 34-fold and *PAX6* at 2.7-fold greater than the housekeepers. Whereas *PAX5* and *PAX8* were expressed 0.1-fold and 0.6-fold to that of the housekeepers, respectively (**Figure 4**). As a comparison we investigated *PAX* gene expression in the melanoma cell line UACC62 which had only *PAX8* expression (0.04-fold), and the prostate cancer cell line PC-3 which expressed *PAX6* at 5-fold and *PAX5* and *PAX8* at the same level as housekeepers (1-fold) (**Figure 4**).

### 3.3 EG1 Slows Proliferation in Human Colorectal Cancer Cells

**Figure 5:**
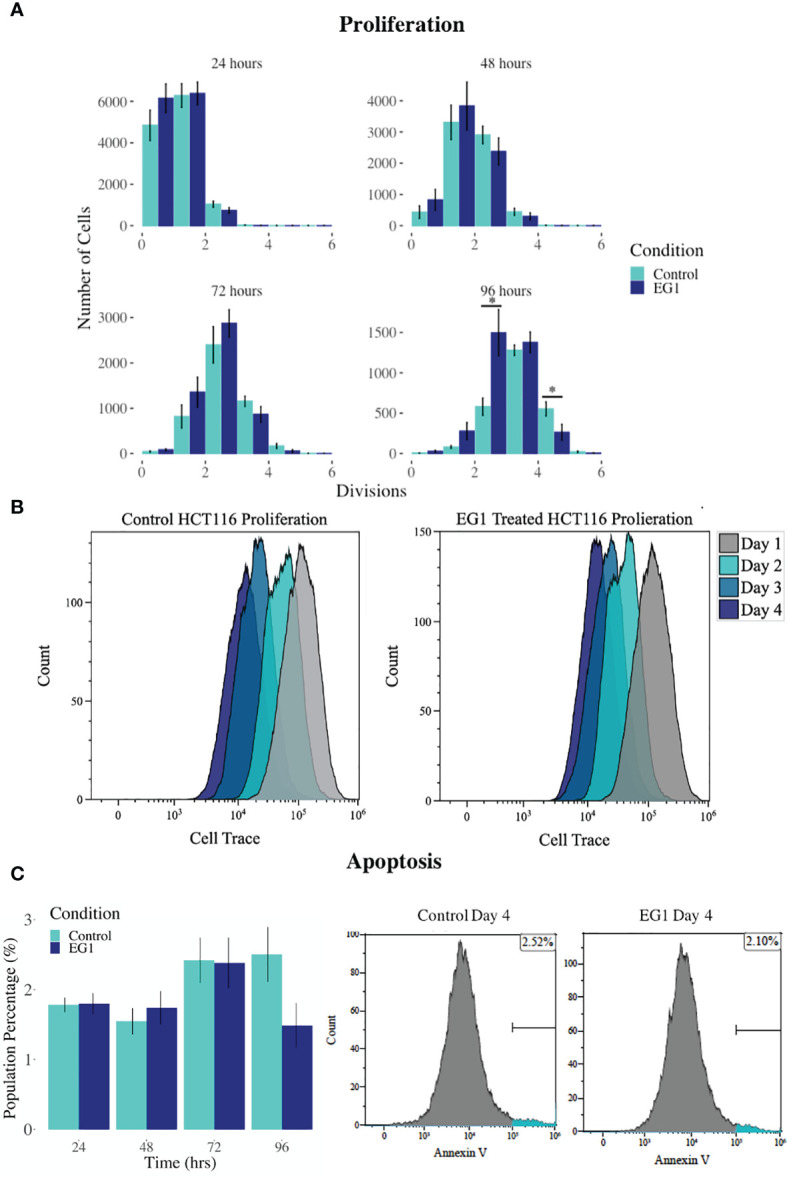
Proliferation and apoptosis in 25 μmol/L EG1 treated and control HCT116 cell cultures. **(A)** A dye dilution flow cytometry assay was performed to determine proliferation in the HCT116 cultures at 24 hours, 48 hours, 72 hours and 96 hours. The population proportion per cellular division was calculated and plotted to show population distributions. At 96 hours the EG1 treated cultures (dark blue) had undergone significantly fewer divisions than the controls (light blue)(*p < 0.05, n=4). **(B)** Flow cytometry data of the dye dilution assay representing the loss of fluorescence in the controls as they divided and retention of fluorescence in the EG1 treated as proliferative events slowed over the 96 hours (n=4). **(C)** Apoptosis in the control and EG1 treated HCT116 cultures measured *via* flow cytometry showed no change in apoptosis over the 96 hours. (n=7).

**Figure 6:**
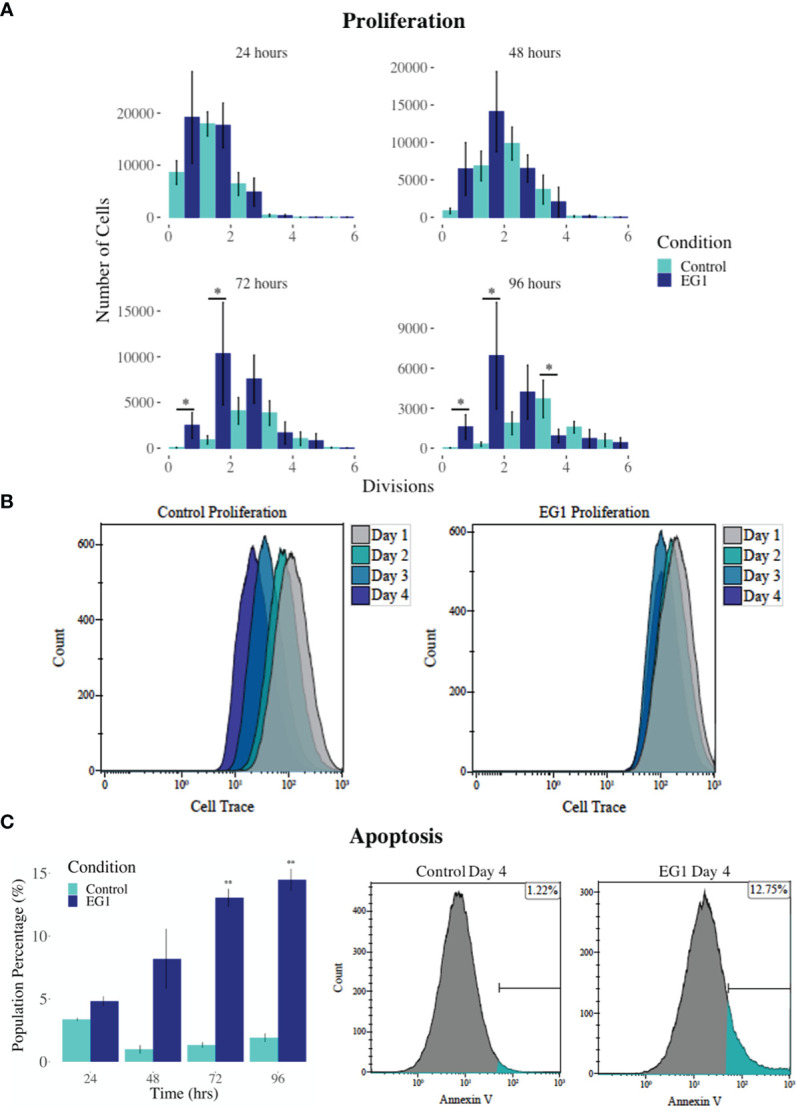
Proliferation and apoptosis in 250 μmol/L EG1 treated and control HCT116 cell cultures. **(A)** A dye dilution flow cytometry assay was performed to determine proliferation in the HCT116 cultures at 24 hours, 48 hours, 72 hours and 96 hours. The population proportion per cellular division was calculated and plotted to show population distributions. At 72 and 96 hours the EG1 treated cultures (dark blue) had undergone significantly fewer divisions than the controls (light blue) (*p < 0.05, n=3). **(B)** Flow cytometry data of the dye dilution assay representing the loss of fluorescence in the controls as they divided and retention of fluorescence in the EG1 treated cultures as proliferation ceased over 96 hours (n=3). **(C)** Apoptosis in the control and EG1 treated HCT116 cultures measured *via* flow cytometry showed a significant increase in apoptosis after 72 and 96 hours. (** p < 0.01, n=3).

PAX proteins have been shown to be involved in colorectal cancer cell proliferation, therefore we hypothesized that inhibition of PAX function by EG1 may have an impact on the proliferative rate of HCT116 cells *via* the disruption of PAX-DNA binding and consequently gene transcription. To address this, we used a dye dilution CellTrace assay to enable the investigation of EG1’s effect on proliferation over time in HCT116 cultures (**Figure 5B**). Following the treatment of these cells with 25 μmol/L EG1, we observed differences in median peak fluorescence between days one and four in the control populations when compared to the EG1 treated populations (**Figures 5A, B**). These data suggest that there was a reduction in the proliferative potential of the EG1 treated cells. Specifically, at 72 hours 25% fewer EG1 treated cells had continued into their third division, while 40% more cells had remained in their first division when compared to the controls. After 96 hours, 53% more control cells than EG1 treated cells that had reached their fourth division, and a significant 71% more control cells than treated cells had undergone five divisions (**Figures 5A, B**). When exposing the HCT116 cells to EG1 at a 10-fold concentration, i.e 250 μmol/L, to produce an exaggerated effect, we found that the EG1 treated cells ceased proliferation after 48 hours, with 65% fewer treated cells entering their third and fourth division than the control cells. Additionally, a significant 83% more control cells than the EG1 treated cells carried on into their fifth division after 96 hours of treatment (**Figure 6A**).

**Figure 7:**
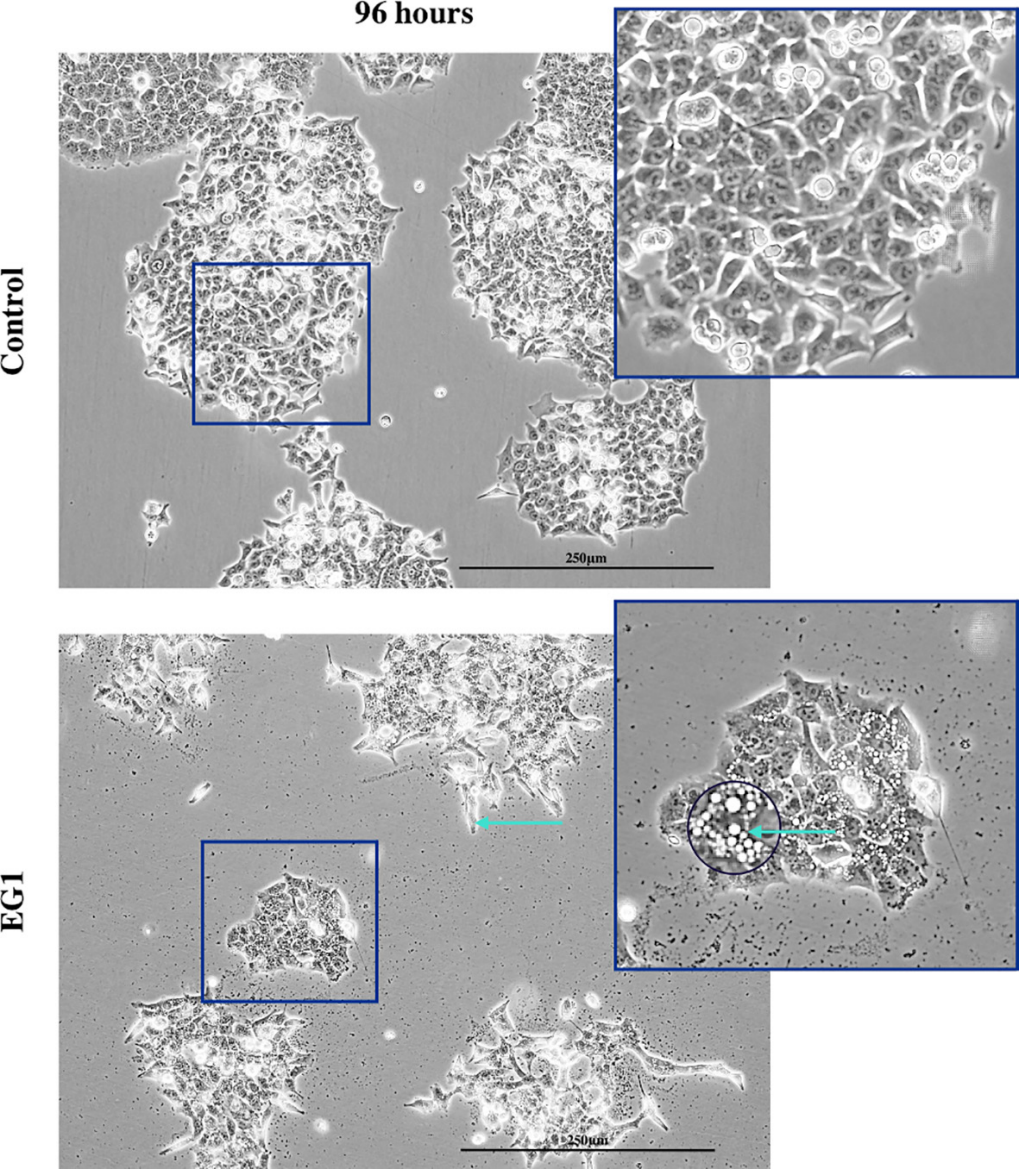
Light microscopy imaging of the control and EG1 treated cultures. Light microscopy image of the control and EG1 treated cultures after 96 hours. The EG1 treated cultures had visibly less growth with some having an elongated phenotype and membrane blebbing (green arrow), an apoptotic feature of epithelial cells.

In addition to proliferation, we investigated apoptosis in the EG1 treated and control cultures to determine if the cessation of cell division was associated with an increase in apoptosis. Over four days 25 μmol/L EG1 treatment had no observable effect on apoptosis occurring in human colon cancer cells (**Figure 5C**). Overall, the highest average population percentages of apoptosis occurred on day four with 2.5% in the control cells and 2.1% in the EG1 treated (**Figure 5C**), although this was not significant. In contrast, at 250 μmol/L, EG1 treatment caused significantly (p<0.05) more HCT116 cells to exhibit apoptosis at 72 and 96 hours (**Figures 6A-C**), which was likely due to the inability of these cells to continue dividing, and therefore exited the cell cycle resulting in cell death. This apoptosis was visualised *via* light microscopy with the cells exhibiting elongation and membrane blebbing (**Figure 7**), which indicates that three days of treatment with 250 μmol/L EG1 is sufficient in inhibiting the anti-apoptotic capability of PAX proteins in these cells (27).

To determine whether EG1 inhibits PAX5, PAX6 and PAX8 proteins the PC-3 prostate cancer cell line was also treated with 25 μmol/L EG1 to observe any effect on proliferation or apoptosis. There was little to no effect of 25 μmol/L EG1 treatment observed in the PC-3 cells while measuring both proliferation and apoptosis over 96 hours (**Supplementary Figure 12.11**). Regardless of the expression of PAX5, PAX6 and PAX8 being higher in the PC-3’s than the HCT116’s, these cells express PAX genes at relatively low levels overall, and thus the impact of EG1 treatment is likely to be more exaggerated in the HCT116’s (**Figure 4**).

### 3.4 EG1 Treatment Modifies Cell Cycle Progression in Human Colorectal Cancer Cells

**Figure 8:**
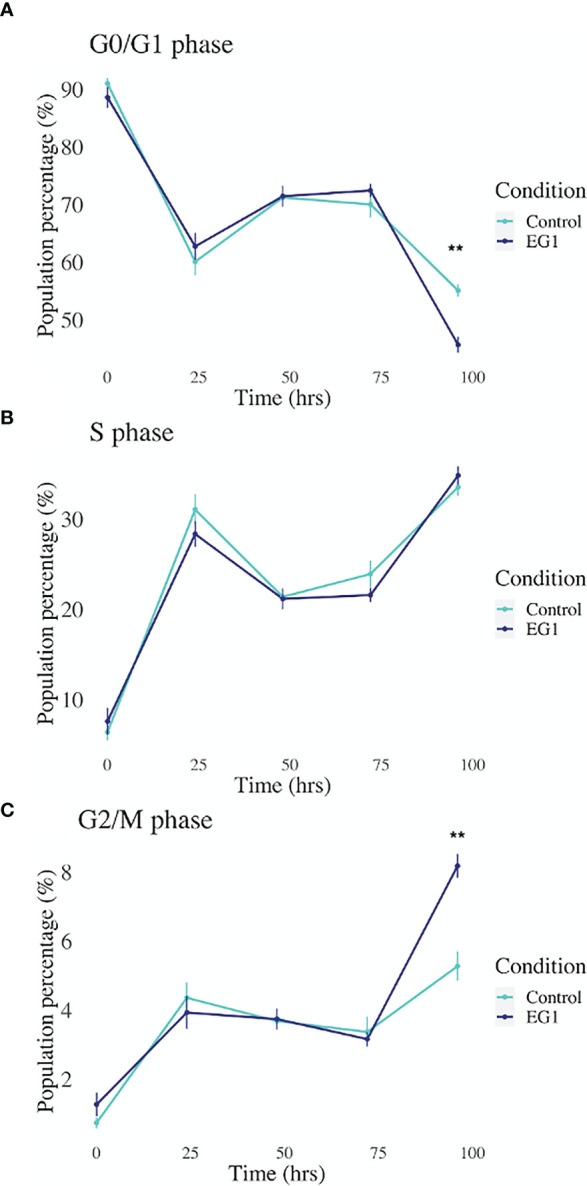
Cell cycling by propidium iodide detection in control and 25 μmol/L EG1 treated HCT116’s. Cell cycling of EG1 treated and control HCT116 cells was measured by propidium iodide (PI) staining and flow cytometry to identify the impact EG1 has on these cells to move through each cell cycle phase (G0/G1, S and G2/M). **(A)** EG1 had no impact on the percent of HCT116 cells in the G0/G1 phases between 24 hours and 72 hours. However, at 92 hours significantly fewer treated cells than controls were detected in this phase (**p < 0.05, n=6). **(B)** EG1 treatment over 96 hours seems to have caused no change in the ability of the HCT116 cells to enter the S phase (n=6). **(C)** The same pattern was observed in the G2/M phases as the G0/G1 phase where between 24 hours and 72 hours there was no difference between the EG1 treated and control populations, yet at 92 hours there were significantly more treated cells in the G2/M phases than the controls. (**p < 0.05, n=6).

Expression of the PAX transcription factors has been linked to differentiation and proliferation during embryonic development, and additionally, with PAX2, PAX5 and PAX8 (subgroup II) have been shown to facilitate tumorigenesis (8, 27). Thus, we investigated whether PAX transcription factors play a role in cell cycling in HCT116 cells after inhibition by 25 μmol/L EG1. Cell cycling in these cultures was measured using flow cytometry, with detection of propidium iodide (PI) staining intensity which is determined by the amount of DNA per cell to identify cell cycle phase (G0/G1, S or G2/M). Data obtained from this indicate that between 24 hours and 72 hours there was no observable difference in the percentage of cells within each phase of the cell cycle when comparing the EG1 treated and the control HCT116 cell cultures (**Figure 8**). However, the inhibition of PAX by EG1 was shown to cause a significant (9.1%) reduction of cells in the G0/G1 phase, and a significant (2.3%) increase of cells in the G2/M phase after 96 hours of EG1 treatment when compared to the control populations (**Figure 8**). Therefore, these data suggest that EG1 treatment causes the cells to become blocked in the G2/M phases, with the likelihood that the HCT116’s fail to enter the G0/G1 phases after 72 hours (**Figure 8**). The inability of these cells to complete the G0/G1 phase may be due to PAX’s involvement in the regulation of cyclin D1, a protein which enables cells to move through the G1 phase (7).

### 3.5 The Impact of EG1 on Gene Expression

**Figure 9:**
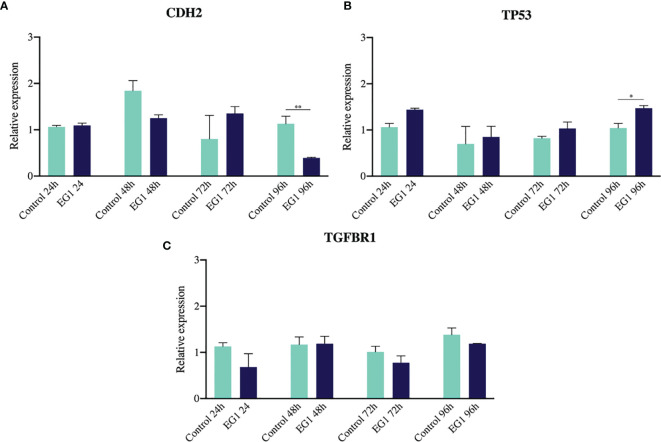
Gene expression of *CDH2*, *TP53* and *TGFBR1 via* RT-qPCR. **(A)** N-cadherin showed a reduction in gene expression on days three and four of treatment, consistent with previous evidence that PAX2 siRNA-mediated knockdown inhibits N-cadherin (*CDH2*) expression (n=3). **(B)** The tumour suppressor *TP53* had increased expression after four days of EG1 treatment, which is hypothesized to be due to *TP53* being normally transcriptionally repressed by PAX2, PAX5 and PAX8 regulation (n=3). **(C)** There was no change in *TGFBR1* expression between the control and EG1 treated populations over 96 hours (n=3). (*p < 0.05, **p < 0.001).

To further substantiate the inhibitory effect of EG1 on PAX2 regulated downstream gene expression we measured the abundance of three target genes which have previously been associated with PAX transcription factor binding (20, 28, 29). To do this we analysed the expression of *CDH2*, *TP53* and *TGF-βR1* using RT-qPCR following treatment of HCT116 cells with 250 μmol/L EG1. We identified significant changes in expression between control and treated cells after 96 hours in both *CDH2* and *TP53* (**Figure 9**). A significant decrease in *CDH2* expression with EG1 treatment is consistent with PAX2’s role in positively regulating *CDH2* expression, as shown by the effect of *PAX2* knockdown causing a reduction of *CDH2* mRNA levels (20, 30). Additionally, we observed an increase in *TP53* expression, consistent with the evidence that PAX proteins (PAX2, PAX5 and PX8) mediate repression of *TP53* expression (29). We also measured the expression of *TGF-βR1* although we saw no change in the expression of this gene over the 96 hours (**Figure 9**).

## 4 Discussion

In this study we provide the first detailed description of chemical synthesis for the compound EG1 (*Results* and **Supplementary Methods**). Initial attempts to prepare EG1 by other pathways resulted in the formation of synthetic intermediates that were either insoluble or unreactive, and these could not be taken through to EG1. By modifying the order in which the aromatic moieties were coupled together, these intractable intermediates could be avoided thus providing a viable route to EG1.

Functional analysis of the synthesized EG1, using the CellTrace assay, following treatment of colorectal carcinoma cells (HCT116) with 25 μmol/L EG1 revealed that this compound is able to slow cell proliferation, and resulted in fewer cell divisions than the control population after four days of treatment. EG1 has previously been shown to inhibit functional transactivation by several PAX proteins, namely PAX2, PAX5 and PAX8, of a luciferase reporter construct (22). However, HCT116 cells express low levels of PAX5 and PAX8, and therefore it is likely that EG1 is targeting PAX2 in these cells. Likewise, two downstream targets of PAX2, *CDH2* and *TP53*, both showed altered gene expression patterns which is consistent with the downregulation of PAX2 transcriptional activity. It is also possible that EG1 has an inhibitory effect on PAX6 as this protein has sequence homology with PAX2 and is expressed in HCT116 cells (**Figure 4**). Furthermore, differing levels of PAX6 have been shown to regulate cell cycle progression in colorectal cancer cells with high levels leading to an increase in cell proliferation (31) through the modulation of PI3K/AKT signalling (32). It is therefore plausible that EG1 could also affect proliferation *via* PAX6.

We found that EG1 treatment inhibited the expression of PAX2 target genes. Importantly, there was a decrease in the expression of *CDH2* which has been shown to be positively correlated with PAX2 activity, and is important in signalling stem cell differentiation and has been linked to cancer metastasis (33). Additionally, the increase observed in the expression of *TP53* at 72 hours and 96 hours in the EG1 treated cells may have also had an effect on cell proliferation as *TP53* plays a key role as a cell cycle regulator and thus could also affect proliferation in these cells (34). Therefore, EG1 treatment inhibits binding of PAX in the *TP53* promoter, which enables *TP53* to be upregulated, potentially contributing to a loss of cell death in the HCT116’s.

PAX8, another PAX family member, is commonly overexpressed in cancers (8) and has previously been found to play a pro-proliferative and anti-apoptotic role in high grade serous carcinoma *via* activation of mutant tumour protein p53 (TP53) (35), thus inhibiting the normal role of p53 as a tumour suppressor. Typically the activation of all three vertebrate orthologues (PAX2, PAX5 and PAX8) reduces expression of wild-type p53 (29), with PAX5 and PAX8 having been shown to do so by directly repressing p53 transcription. The resultant loss of p53 enables damaged cells to move through cell cycle checkpoints, and as a consequence reduces division time and increases the speed of cell cycling. We propose that EG1 acts to inhibit DNA binding of aberrantly elevated PAX2 levels in cancer cells, thus enabling an increase in normal *TP53* expression (**Figure 9**). This hypothesis is supported by our observation that EG1 treatment of colorectal carcinoma cells expressing PAX2 led to an increase in *TP53* mRNA expression (**Figure 9**).

**Figure 10:**
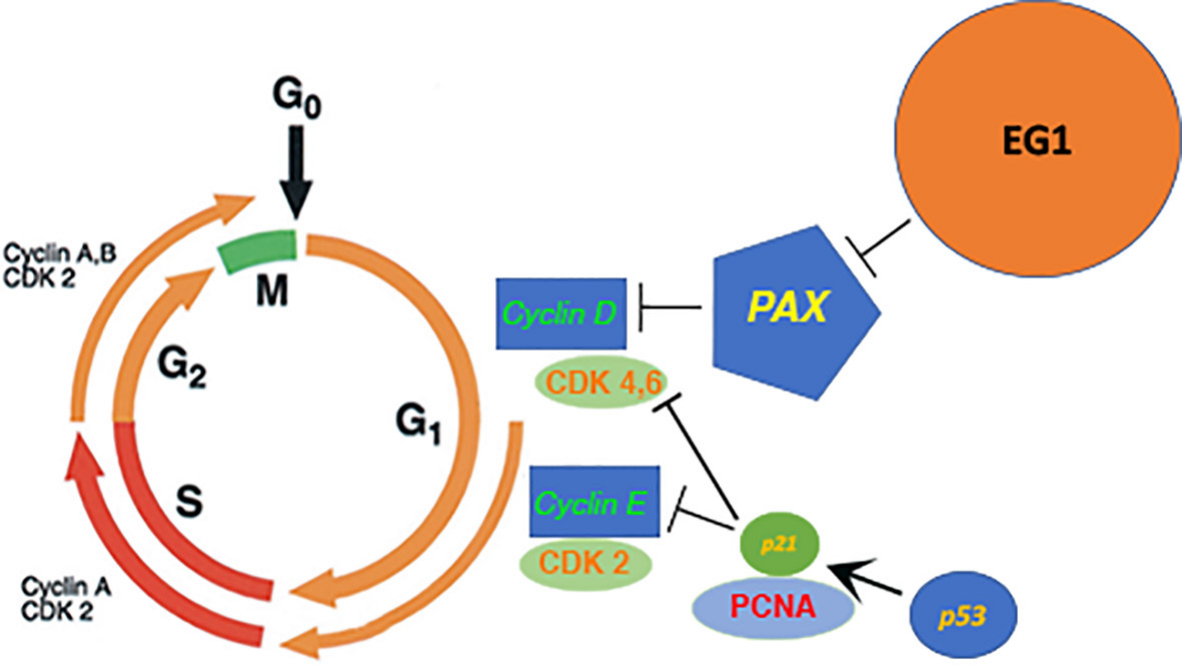
Proposed model of the downstream effect that EG1 has on cell cycling *via* PAX inhibition. The EG1 compound is expected to block the interaction between the PAX transcription factors and DNA which causes a loss of polymerase recruitment to the site and a reduction of PAX regulated gene transcription resulting in the disruption of cyclin dependant kinase (CDK) activation. A reduction of CDK activity will cause a loss of cell signalling and thus interrupts the normal cell cycling process.

Following 25 μmol/L EG1 treatment there was no significant increase in apoptosis, suggesting that the reduction in HCT116 cell number observed was not due to an increase in the number of cells that died. Comparatively, when treated with 250 μmol/L EG1 we observed a significant increase in the percentage of apoptosed cells compared to the controls which suggests that this concentration of the compound is toxic to these cells. We hypothesize that this is caused by an inability of these cells to continue dividing and thus are exiting the cell cycle. Interestingly, PAX2 has previously been shown to regulate the *CCND1* gene (7) which encodes cyclin D1, a protein that normally acts to enable cellular progression into and through the G1 and S phases by regulating cyclin dependent kinases (CDKs), specifically CDK4 and CDK6 which are active in the G1 phase (**Figure 10**). Importantly, cyclin D1 dysregulation has previously been associated with a number of cancers and is linked to a poorer prognosis; however, there is yet to be an approved therapy which targets this protein.

Overall, we have successfully synthesized the EG1 compound and shown its ability to inhibit PAX transcription factor-mediated downstream gene regulation in the HCT116 human colorectal carcinoma cells, identified by changes in the expression of downstream genes. Additionally, the inhibition of PAX by EG1 had an impact on the proliferation of the HCT116 cells. We hypothesize that this compound will have similar effects in other cancer cell types which also aberrantly express PAX proteins at a high level, particularly PAX2, and potentially PAX5, PAX6 or PAX8. Due to the developmentally regulated and tissue-specific nature of *PAX* gene expression it is possible that inhibiting PAX transcription factors would have minimal toxic effects in healthy adult tissue, as PAX transcription factors are primarily expressed, and critically required during organ development in the fetus. Follow-up studies of EG1 treatment in other disease cell types, and further elucidation of the downstream cell cycle pathway changes will inform future studies and potential applications of this compound in treating diseases associated with over-expression of *PAX* genes.

## Data Availability Statement

The original contributions presented in the study are included in the article/**Supplementary Material**. Further inquiries can be directed to the corresponding authors.

## Author Contributions

ME, CS, and AW conceived and designed the experiments. LM performed the experiments and carried out data analysis. ME, CS, AW, SM, and LM wrote the manuscript and provided critical analysis. JK, JW, and DL developed and carried out the chemical synthesis and analysis of EG1, and contributed to the writing and review of the manuscript. All authors contributed to the article and approved the submitted version.

## Funding

This research was supported by a NZ Lottery Health grant. JW was awarded the University of Otago Summer Studentship Scholarship. SM and AW are supported by the Maurice Wilkins Centre for Molecular Biodiscovery.

## Conflict of Interest

The authors declare that the research was conducted in the absence of any commercial or financial relationships that could be construed as a potential conflict of interest.

## Publisher’s Note

All claims expressed in this article are solely those of the authors and do not necessarily represent those of their affiliated organizations, or those of the publisher, the editors and the reviewers. Any product that may be evaluated in this article, or claim that may be made by its manufacturer, is not guaranteed or endorsed by the publisher.
