# Supplementary material for: Chemical Synthesis of the PAX Protein Inhibitor EG1 and Its Ability to Slow the Growth of Human Colorectal Carcinoma Cells
Source: Front Oncol. 2021 Oct 13;11:709540. doi: 10.3389/fonc.2021.709540 (PMC8549845; doi:10.3389/fonc.2021.709540)
Supplement: Supplementary file 1 [file DataSheet_1.pdf]

Supplementary Material

11 Supplementary methods

11.1 Previous reporting on EG1

An exact structure search for EG1 performed using SciFinder on the 14th of August 2020 gave only the paper by Dressler, G. R. *et al. ACS Chemical Biology* **2017**, *12*, 724-734 which did not report a chemical synthesis for EG1.

11.2 Chemical synthesis of EG1

Experiments requiring anhydrous conditions were performed under a nitrogen or argon atmosphere using apparatus heated and dried under vacuum, unless stated otherwise.

Anhydrous dichloromethane (CH_2Cl_2) and tetrahydrofuran (THF) were dried using the PURE SOLV MD-6 solvent purification system. All other reagents were purchased as analytical or reagent grade and used without further purification. Aqueous solutions of sodium chloride (NaCl), sodium bicarbonate ($NaHCO_3$) and ammonium chloride (NH_4Cl) were saturated. Reactions performed at room temperature (rt) were carried out at approximately 20 °C and reaction temperatures at 0 °C were performed using a water/ice cooling bath mixture. Reactions were monitored by thin layer chromatography (TLC) carried out on 0.2 mm Kieselgel F254 (Merck) silica gel plates using UV light as a visualising agent and then stained and developed with heat using either vanillin in ethanolic sulfuric acid, ammonium heptamolybdate and cerium sulfate in aqueous sulfuric acid, or potassium permanganate and potassium carbonate in aqueous sodium hydroxide. Separation of mixtures was performed by flash chromatography using 0.063–0.1 mm silica gel with the indicated eluent. Melting points were determined using either a DigiMelt SRS hot stage or a ThermoFisher Scientific IA9000 Digital melting point apparatus and are uncorrected. Infrared spectra were recorded on a Bruker Optics Alpha FT-IR spectrometer with a diamond Attenuated Total Reflectance (ATR) top plate. No sample preparation was required. Absorption peaks are reported as wavenumbers (v, cm^{-1}).

NMR spectra were recorded on a Varian 400-MR spectrometer operating at 400 MHz for ^1H nuclei and 100 MHz for ^{13}C nuclei at 25 °C, or a Varian 500 MHz AR Premium Shielded Spectrometer operating at 500 MHz for ^1H nuclei and 125 MHz for ^{13}C nuclei at 25 °C. ^1H NMR chemical shifts are reported in parts per million (ppm) relative to the chloroform ($CDCl_3$, δ 7.26), methanol (CD_3OD, δ 3.31), or dimethylsulfoxide (DMSO-*d6*, δ 2.50) peak. ^1H NMR values are reported as chemical shifts δ, multiplicity (s, singlet; d, doublet; t, triplet; q, quartet; dd, doublet of doublets; td, triplet of doublets; m, multiplet), coupling constant (J, Hz), relative integral, and assignment. Coupling constants were taken directly from the spectra. ^{13}C NMR chemical shifts are reported in ppm relative to the chloroform ($CDCl_3$, δ 77.0), methanol (CD_3OD, δ 49.0), or dimethylsulfoxide (DMSO-*d6*, δ 39.5) peak. ^{13}C NMR values are reported as chemical shifts δ and assignment. Assignments were made with the aid of gCOSY, gHSQC, and gHMBC experiments.

Mass spectra were recorded on a Bruker micrOTOF-Q II mass spectrometer by electrospray ionisation in positive and negative mode. High-resolution mass spectra (HRMS) were obtained with a nominal resolution of 5,000 to 10,000.

11.3 LC-MS analysis

Mass spectrometry grade acetonitrile (CH_3CN) and water (H_2O) were purchased from Thermofisher Scientific. Mass spectrometry grade formic acid was purchased from Sigma-Aldrich. RP-LC-MS analyses were conducted on an analytical RP-HPLC (Shimadzu LC–20AD equipped with an SPD-20A UV detector [210 and 254 nm] and a Shimadzu LC-MS-2020 Liquid Chromatograph Mass Spectrometer operating in positive or negative ion mode) using a Phenomenex Prodigy column (C–18, 5 μm, 3.00 × 250 mm) at 0.5 mL/min and heated to 40 °C. The solvent system for LC purposes was a mixture of A (0.1% formic acid in H_2O) and B (0.1% formic acid in CH_3CN).

11.4 Methyl 2-(4'-nitrobenzamido)benzoate (3)

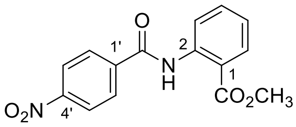

Thionyl chloride (940 μL, 11.99 mmol) was added to a suspension of 4-nitrobenzoic acid (**1**) (1 g, 5.98 mmol) in anhydrous CH_2Cl_2 (30 mL) at room temperature (rt). A catalytic quantity of DMF (two drops) was added to the reaction mixture at rt, effervescence resulted and the reaction mixture was heated to reflux. After six hours the reaction mixture had dissolved to a clear solution which was allowed to cool to rt and concentrated *in vacuo* to afford the acid chloride as a yellow solid. Without further purification, the acid chloride was then immediately dissolved in anhydrous CH_2Cl_2 (25 mL). A solution of methyl 2-aminobenzoate (**2**) (1.07 g, 7.06 mmol) and anhydrous DIPEA (2.2 mL) in anhydrous CH_2Cl_2 (20 mL) was cooled to 0 ˚C and the acid chloride solution was added over five minutes. The reaction mixture was then heated to reflux for five hours and then cooled to 0˚C. 1 M aqueous HCl (50 mL) was slowly added and the aqueous phase was extracted with CH_2Cl_2 (3 x 50 mL). The combined organic extracts were washed sequentially with 1 M aqueous HCl (50 mL) and H_2O (2 x 50 mL), then dried over Na_2SO_4 and concentrated *in vacuo* to obtain a yellow solid. Purification by crystallisation from a mixture of hot CH_2Cl_2 (30 mL) and petroleum ether (20 mL) gave yellow crystals which were isolated by Buchner filtration to afford the title compound **3** (1.22 g, 68%). M.p. 196–198 °C (Lit. M.p.[1] 197–199 °C); ^{1}H NMR (400 MHz, $CDCl_3$) δ 3.99 (s, 3H, $CO_2C\underline{H_3}$), 7.19 (m, 1H, 5–H), 7.63–7.67 (m, 1H, 4–H), 8.12 (dd, $^{3}J_{2,3}$ 8.0, $^{4}J_{2,4}$ 1.7 Hz, 1H, 6–H), 8.20–8.24 (m, 2H, 2'–H and 6'–H), 8.37–8.40 (m, 2H, 3'–H and 5'–H), 8.89–8.92 (m, 1H, 3–H), 12.28 (br s, 1H, N$\underline{H}$–2–H); ^{13}C NMR (100 MHz, $CDCl_3$) δ 52.7 (CH_3, $CO_2\underline{C}H_3$), 115.3 (C_{quat}, C–1), 120.4 (CH, C–3), 123.4 (CH, C–5), 124.0 (CH, C–3' and C–5'), 128.5 (CH, C–2' and C–6'), 131.1 (CH, C–6), 135.0 (CH, C–4), 140.4 (C_{quat}, C–1'), 141.2 (C_{quat}, C–2), 141.2 (C_{quat}, C–6), 149.8 (C_{quat}, C–4'), 163.5 (C_{quat}, C–1'–$\underline{C}$O), 169.2 (C_{quat}, $\underline{C}O_2CH_3$). The NMR data was consistent with literature values.[1,2]

11.5 4'-((2-(Methoxycarbonyl)phenyl)carbamoyl)benzenaminium acetate (4)

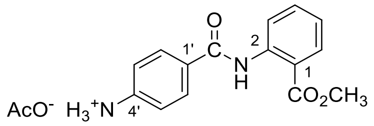

Palladium on charcoal (10 % weight, 177 mg, 0.17 mmol) was added to a suspension of **3** (0.5 g, 1.67 mmol) in a mixture of $AcOH:CH_2Cl_2:EtOAc$ (1:10:30, 41 mL) and flushed with N_2 gas for 10 minutes. H_2 gas (1 atm) was then introduced and the reaction mixture heated to 35–40 ˚C. After 22 hours the reaction mixture was flushed with N_2 gas for 10 minutes and the mixture was filtered through Celite. The Celite was washed with CH_2Cl_2 (75 mL) and the organic filtrate concentrated *in vacuo* to obtain the *title compound* **4** as a white solid (432 mg, 79%). M.p. 185–187 °C; IR (ATR) ν_{max}/cm^{-1} 3476, 3366, 1691, 1651, 1602, 1585, 1509, 1431, 1296, 1254, 1179, 756; ^1H NMR (400 MHz, CD_3OD) δ 3.98 (s, 3H, $CO_2C\underline{H_3}$), 6.72–6.76 (m, 2H, 3'–H and 5'–H), 7.15 (ddd, $^3J_{3,2}$ 8.0, $^3J_{3,4}$ 7.3, $^4J_{3,5}$ 1.2 Hz, 1H, 5–H), 7.60 (ddd, $^3J_{4,5}$ 8.7, $^3J_{4,3}$ 7.3, $^4J_{4,2}$ 1.7 Hz, 1H, 4–H), 7.76–7.80 (m, 2H, 2'–H and 6'–H), 8.10 (dd, $^3J_{2,3}$ 8.0, $^4J_{2,4}$ 1.7 Hz, 1H, 6–H), 8.76 (dd, $^3J_{5,4}$ 8.7, $^4J_{5,3}$ 1.2 Hz, 1H, 3–H); ^{13}C NMR (100 MHz, CD_3OD) δ 53.0 (CH_3, $CO_2\underline{C}H_3$), 114.8 (CH, C–3' and C–5'), 116.7 (C_{quat}, C–1), 121.4 (CH, C–3), 122.9 (C_{quat}, C–4'), 123.6 (CH, C–5), 130.2 (CH, C–2' and C–6'), 132.1 (CH, C–6), 135.5 (CH, C–4), 143.1 (C_{quat}, C–2), 154.1 (C_{quat}, C–1'), 167.9 (C_{quat}, C–1'–$\underline{C}$O), 170.4 (C_{quat}, $\underline{C}O_2CH_3$); HRMS (ESI-TOF) *m/z*: $[M + Na]^+$ Calcd for $C_{15}H_{14}N_2NaO_3{}^+$ 293.0895; found 293.0897.

Note: this compound has been prepared as its free base by Hamuro et al.[1]

11.6 Methyl 2-(4'-(2''-methoxybenzamido)benzamido)benzoate (EG1 methyl ester 7)

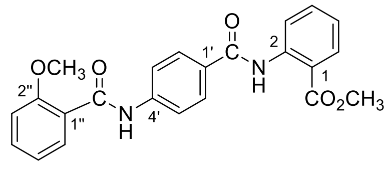

Oxalyl chloride (190 µL, 2.27 mmol) was added to a solution of 2-methoxybenzoic acid **5** (240 mg, 1.54 mmol) in anhydrous CH_2Cl_2 (8 mL) at rt. A catalytic quantity of DMF (two drops) was added to the reaction mixture at rt to result in effervescence. The reaction mixture was stirred at rt for 1.5 hours and then concentrated *in vacuo* to afford acid chloride **6** as a yellow oil. Without further purification, acid chloride **6** was immediately diluted in anhydrous CH_2Cl_2 (7 mL) and cooled to 0 ˚C. In a separate round bottom flask, a solution of amine acetate salt **4** (503 mg, 1.52 mmol) was prepared by the subsequent addition of anhydrous DMF (3 mL), DIPEA (620 µL, 3.56 mmol) and then anhydrous CH_2Cl_2 (12 mL). The resulting solution of amine acetate **4** was then added slowly over five minutes to the cooled solution of acid chloride **6**. The reaction mixture was then allowed to warm to rt and stirred for 69 hours and then concentrated *in vacuo*. The residue was taken up in saturated NH_4Cl (20 mL) and the aqueous phase extracted with CH_2Cl_2 (3 x 50 mL). The combined organic extracts were washed with H_2O (80 mL), then dried over Na_2SO_4 and concentrated *in vacuo* to obtain a yellow oil. Purification was performed first with flash chromatography (CH_2Cl_2 load, 0%, then 35%, then 40 % EtOAc in petroleum ether, then 50% EtOAc in

CH_2Cl_2). The fractions containing the product were combined and concentrated *in vacuo* to afford a yellow solid. This was purified further by trituration from CH_2Cl_2 (20 mL) and petroleum ether (40 mL) to yield the *title compound* **7** as a pale orange-white powder (358 mg, 60%). M.p. 189 °C; R_f (35% EtOAc in petroleum ether) 0.24; IR (ATR) ν_{max}/cm^{-1} 3320, 3255, 2950, 2839, 1698, 1665, 1589, 1530, 1509, 1434, 1295, 1258, 1235, 1179, 1160, 754; ^{1}H NMR (500 MHz, $CDCl_3$) 3.93 (s, 3H, $CO_2C\underline{H_3}$), 4.02 (s, 3H, 2''–$OC\underline{H_3}$), 6.98 (d, $^3J_{3'',4''}$ 10.0 Hz, 1H, 3''–H), 7.06–7.09 (m, 2H, 5–H and 5''–H), 7.44–7.46 (m, 1H, 4''–H), 7.54–7.56 (m, 1H, 4–H), 7.79–7.81 (m, 2H, 3'–H and 5'–H), 8.01–8.04 (m, 3H, 6–H, 2'–H and 6'–H), 8.25 (d, $^3J_{6'',5''}$ 10.0, Hz, 1H, 6''–H), 8.90 (d, $^3J_{3,4}$ 10.0 Hz, 1H, 3–H), 9.98 (s, 1H, N$\underline{H}$–4'–H), 11.99 (s, 1H, N$\underline{H}$–2–H); ^{13}C NMR (125 MHz, $CDCl_3$) δ 52.37, 52.40 (CH_3, $CO_2\underline{C}H_3$), 56.17, 56.20 (CH_3, 2''–$O\underline{C}H_3$), 111.5 (CH, C–3''), 115.0 (C_{quat}, C–1), 119.8 (CH, C–3' and C–5'), 120.2 (CH, C–3), 121.3 (C_{quat}, C–1''), 121.6 (CH, C–5''), 122.3 (CH, C–5), 128.5 (CH, C–2' and C–6'), 129.9 (C_{quat}, C–1'), 130.9 (CH, C–6), 132.5 (CH, C–6''), 133.5 (CH, C–4''), 134.7 (CH, C–4), 141.8 (C_{quat}, C–4'), 141.9 (C_{quat}, C–2), 157.1 (C_{quat}, C–2''), 163.3 (C_{quat}, C–1''–$\underline{C}$O), 165.0 (C_{quat}, C–1'–$\underline{C}$O), 168.9 (C_{quat}, $\underline{C}O_2CH_3$); RP-LC-MS (10% to 100% B over 12.5 min, then 100% B for 6.5 min, 100% to 10% B over 1 min, then 10% B for 3 min), t_R = 13.45 minutes; MS *m/z* (ESI+): 405 ([M + H]$^+$, 100%), 427 ([M + Na]$^+$, 34), 809 ([2M + H]$^+$, 93), 831 ([2M + Na]$^+$, 72); HRMS (ESI-TOF) *m/z*: [M + Na]$^+$ Calcd for $C_{23}H_{20}N_2NaO_5^+$ 427.1264; found 427.1277.

11.7 2-(4'-(2''-Methoxybenzamido)benzamido)benzoic acid (EG1)

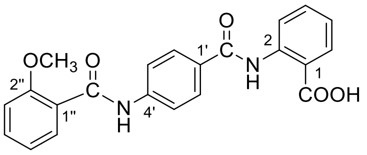

0.5 M aqueous NaOH (1.5 mL, 0.74 mmol) was added to a suspension of EG1 methyl ester (**7**) (100 mg, 0.25 mmol) in THF (5mL) at rt. The reaction mixture was stirred at 30 ºC for 10 minutes to dissolve all solids and then allowed to stir at rt. After 16 hours, the reaction mixture was acidified with 1 M aqueous HCl to pH 1-2 and extracted with EtOAc (3 x 50 mL). The combined organic extracts were washed with H_2O (50 mL), then saturated NaCl (50 mL) and then dried over $MgSO_4$. The volume of the organic extracts was then reduced *in vacuo* to approximately 5-10 mL (but not to dryness) to obtain a white precipitate which was isolated by Buchner filtration. The precipitate was washed with EtOAc (3 x 10 mL) and dried to afford EG1 as a white powder (68 mg, 70%). M.p. 260–262 °C; IR (ATR) ν_{max}/cm^{-1} 3320, 2987, 1671, 1649, 1583, 1534, 1509, 1447, 1256, 1214, 1020, 758, 743, 672, 651; ^{1}H NMR (500 MHz, DMSO-*d6*) 3.91 (s, 3H, 2''–$OC\underline{H_3}$), 7.08 (dd, $^3J_{5'',4''}$ 7.5, $^4J_{5'',3''}$ 0.9 Hz, 1H, 5''–H), 7.19–7.22 (m, 2H, 5–H and 3''–H), 7.53 (td, $^3J_{4'',3''}$ 8.4, $^3J_{4'',5''}$ 7.5, $^4J_{4'',6''}$ 1.8 Hz, 1H, 4''–H), 7.63–7.69 (m, 2H, 4–H and 6''–H), 7.95 (br s, 4H, 2'–H, 3'–H, 5'–H and 6'–H), 8.06 (dd, $^3J_{2,3}$ 7.6, $^4J_{2,4}$ 1.7 Hz, 1H, 6–H), 8.72 (dd, $^3J_{5,4}$ 8.5, $^4J_{5,3}$ 1.2 Hz, 1H, 3–H), 10.45 (s, 1H, N$\underline{H}$–4'–H), 12.16 (s, 1H, N$\underline{H}$–2–H), 13.79 (br s, 1H, COO$\underline{H}$); ^{13}C NMR (125 MHz, DMSO-*d6*) δ 55.9 (CH_3, 2''–$O\underline{C}H_3$), 112.0 (CH, C–3''), 116.3 (C_{quat}, C–1), 119.3 (CH, C–3' and C–5'), 119.8 (CH, C–3), 120.5 (CH, C–5''), 122.8 (CH, C–5), 124.8 (C_{quat}, C–1''), 128.0 (CH, C–2' and C–6'), 129.0 (C_{quat}, C–1'), 129.6 (C_{quat}, C–6''), 131.3 (CH, C–6), 132.3 (CH, C–4''), 134.3 (CH, C–4), 141.3 (C_{quat}, C–2), 142.5 (C_{quat}, C–4'), 156.5 (C_{quat}, C–2''), 164.2 (C_{quat}, C–1'–$\underline{C}$O), 165.1 (C_{quat}, C–1''–$\underline{C}$O), 170.1 (C_{quat}, $\underline{C}$OOH); RP-LC-MS (10% to 100% B over 12.5 min, then 100% B for 2.5 min, 100% to 10% B over 1 min, then 10% B for 3 min), t_R =

11.59 minutes; MS *m/z* (ESI–): 389 ($[M – H]^-$, 100%), 779 ($[2M – H]^-$, 28); HRMS (ESI-TOF) *m/z*: $[M – H]^-$ Calcd for $C_{22}H_{17}N_2O_5^-$ 389.1143; found 389.1157.

12 Supplementary Figures and Tables

12.1 ^{1}H and ^{13}C NMR spectras of novel and unreported compounds

a) ^{1}H NMR spectrum of 4'-((2-(methoxycarbonyl)phenyl)carbamoyl)benzenaminium acetate (4) (400 MHz, CD_3OD).

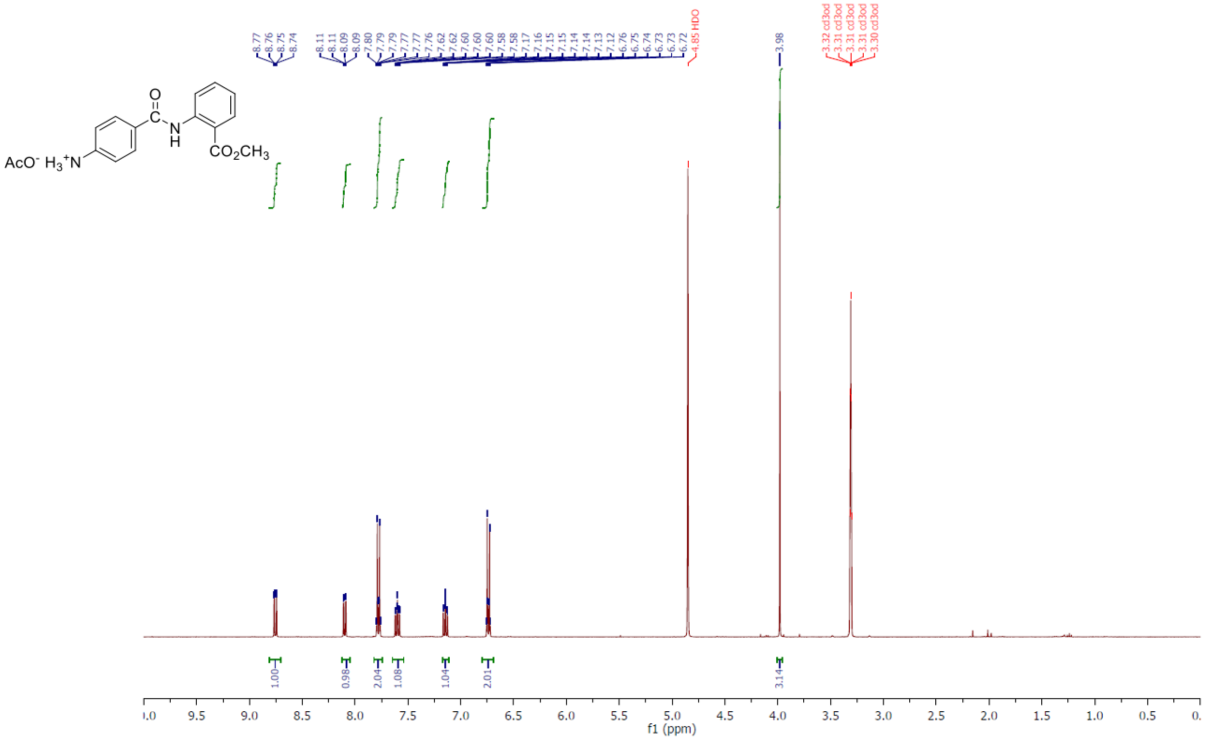

b) ^{13}**C NMR spectrum of 4'-((2 (methoxycarbonyl)phenyl)carbamoyl)benzenaminium acetate (4) (100 MHz, CD$_3$OD).**

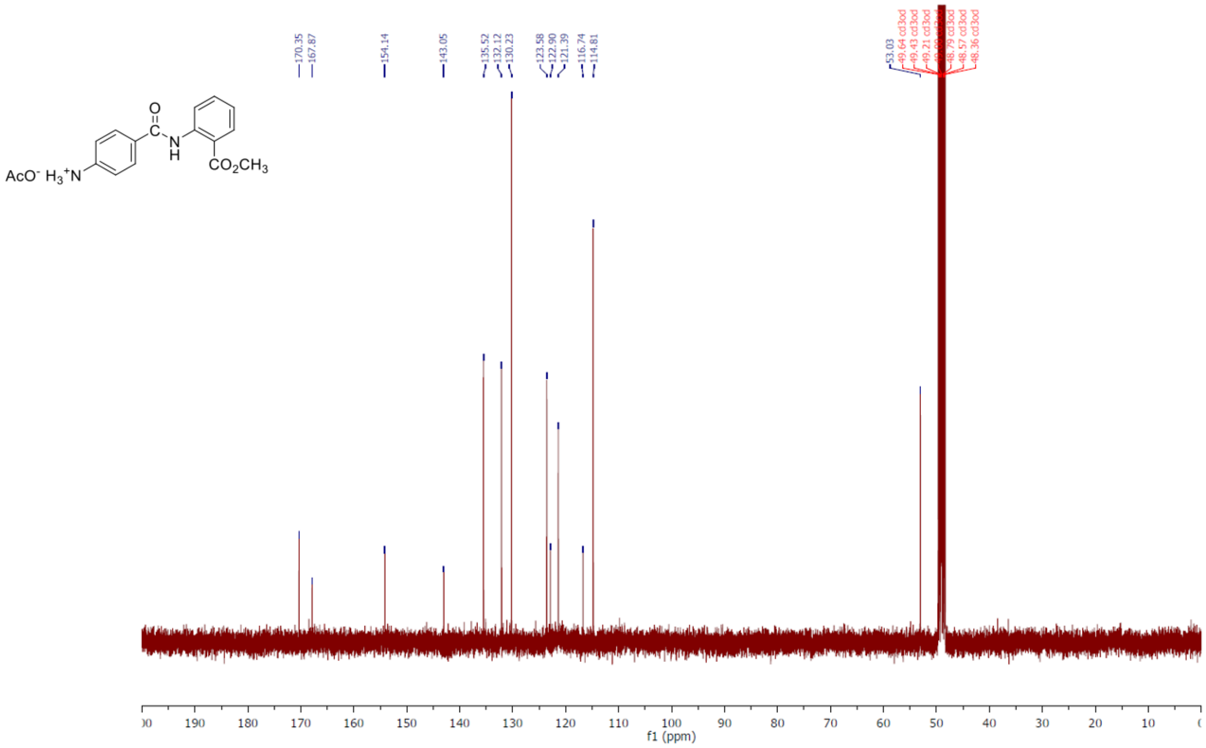

c) ^{1}**H NMR spectrum of methyl 2-(4'-(2''-methoxybenzamido)benzamido)benzoate (EG1 methyl ester 7) (500 MHz, CDCl$_3$).**

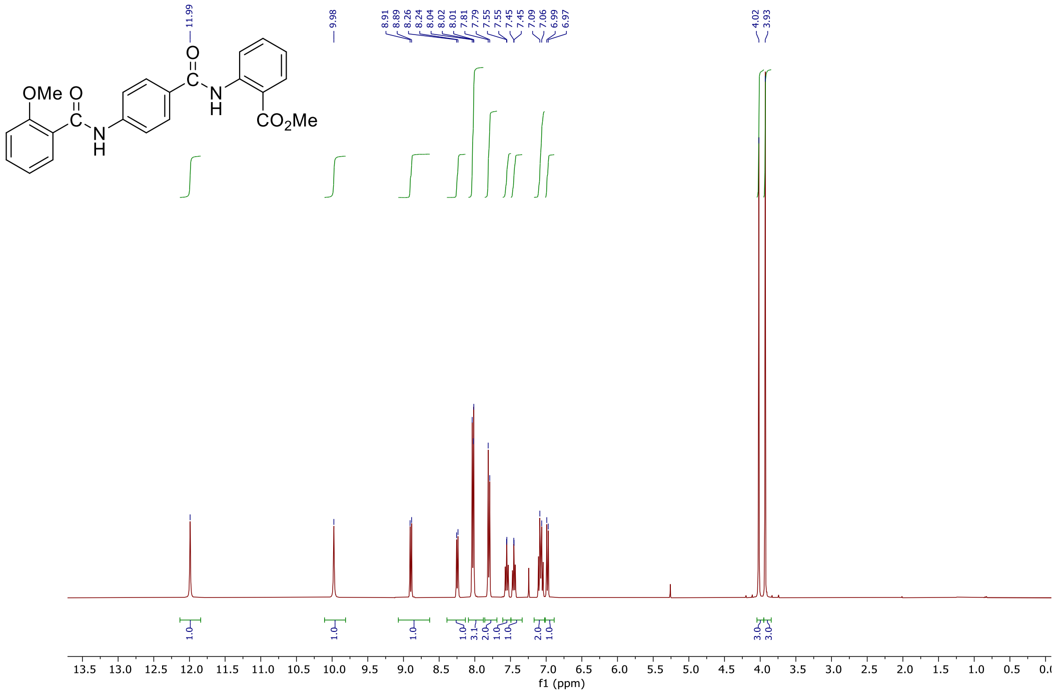

d) ^{13}**C NMR spectrum of methyl 2-(4'-(2''-methoxybenzamido)benzamido)benzoate (EG1 methyl ester 7) (125 MHz, CDCl$_3$).**

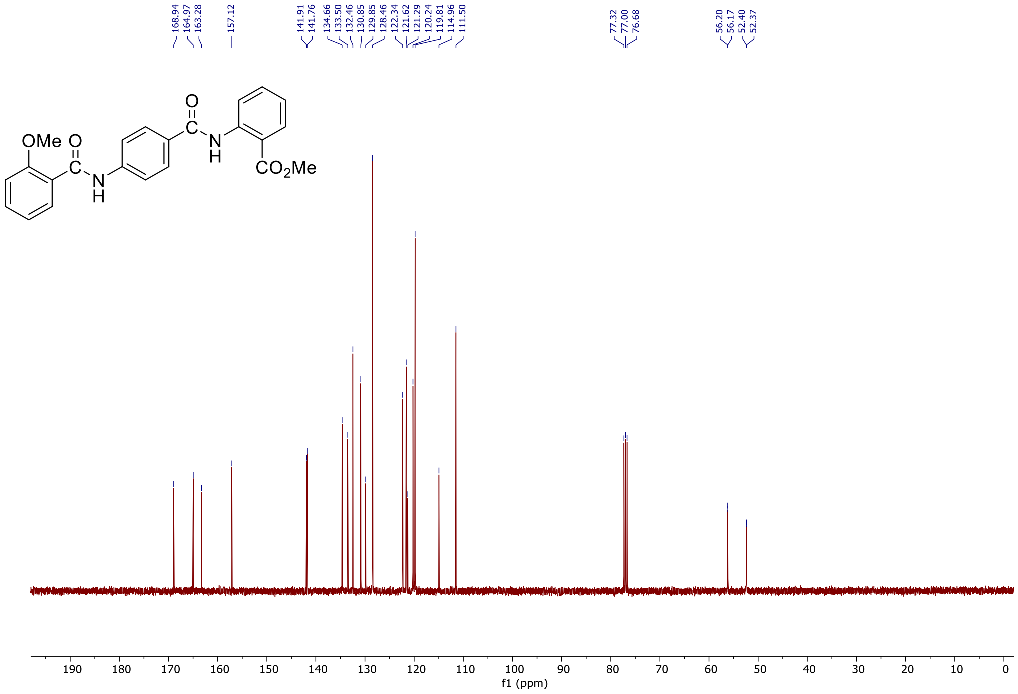

e) ^{1}H NMR spectrum of 2-(4'-(2''-methoxybenzamido)benzamido)benzoic acid (EG1) (500 MHz, DMSO-*d6*).

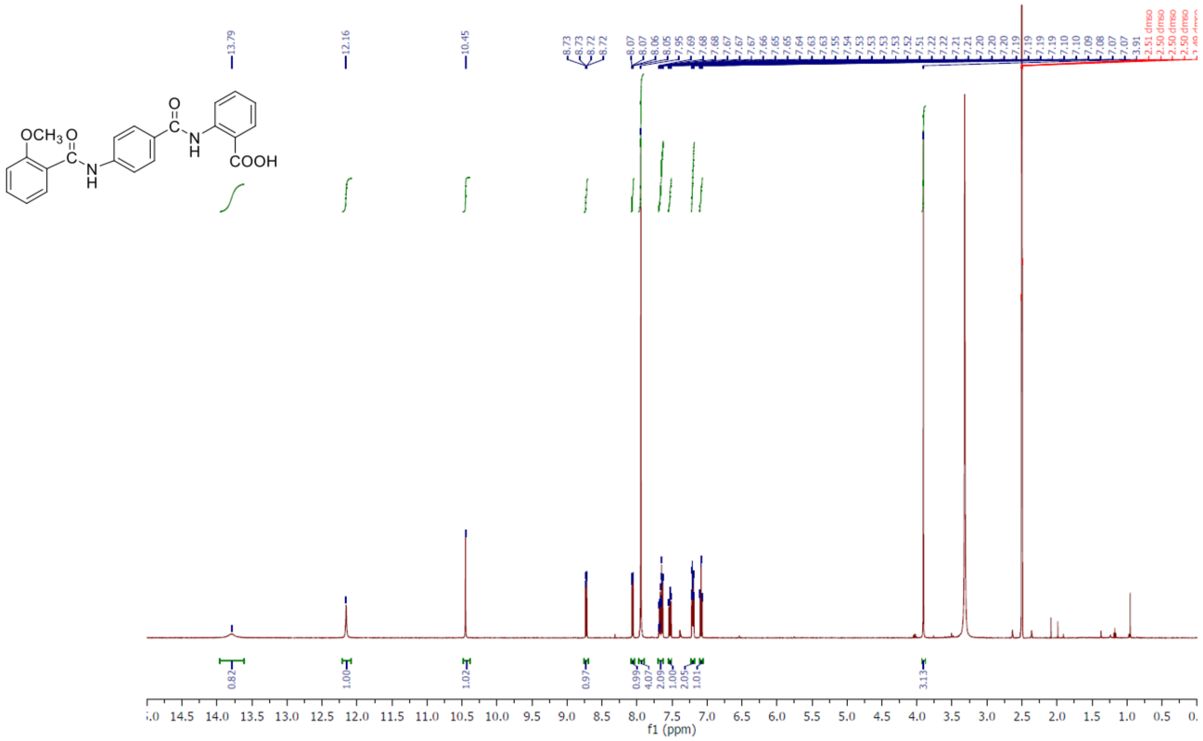

f) ^{13}C NMR spectrum of 2-(4'-(2''-methoxybenzamido)benzamido)benzoic acid (EG1) (125 MHz, DMSO-*d6*).

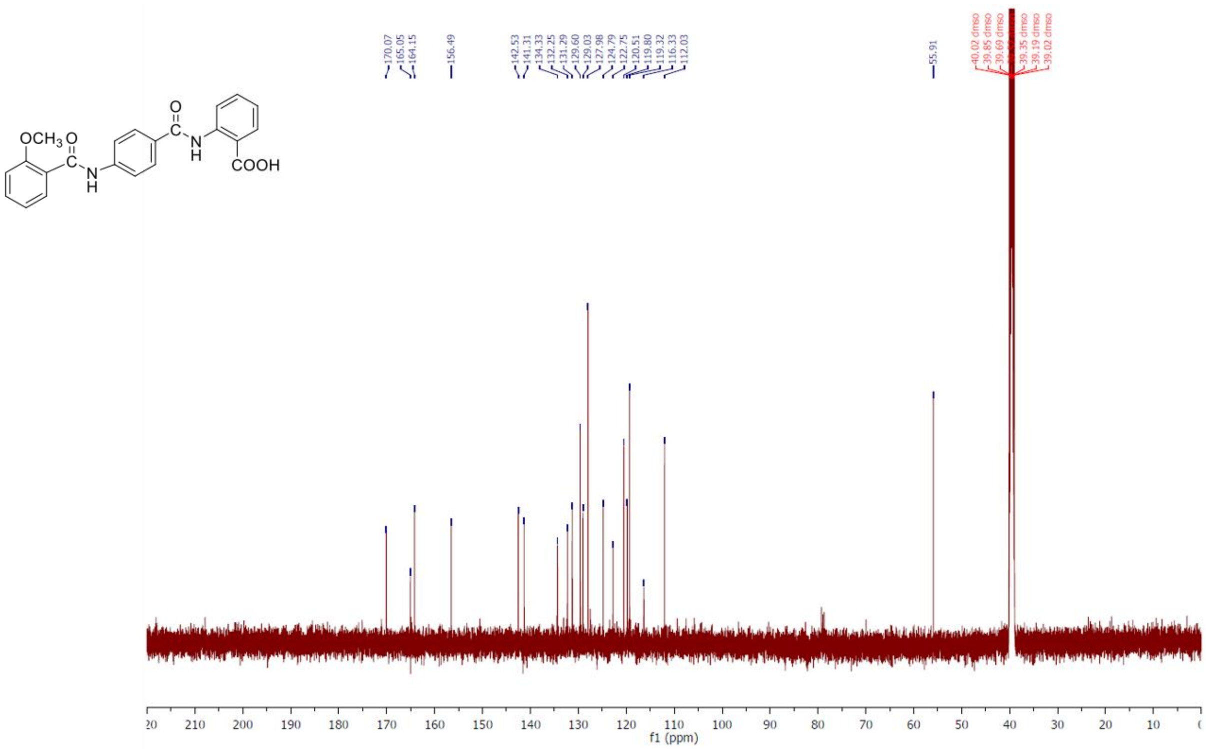

12.2 Standard curve for EG1 by LC-MS using Selective Ion Monitoring (SIM)

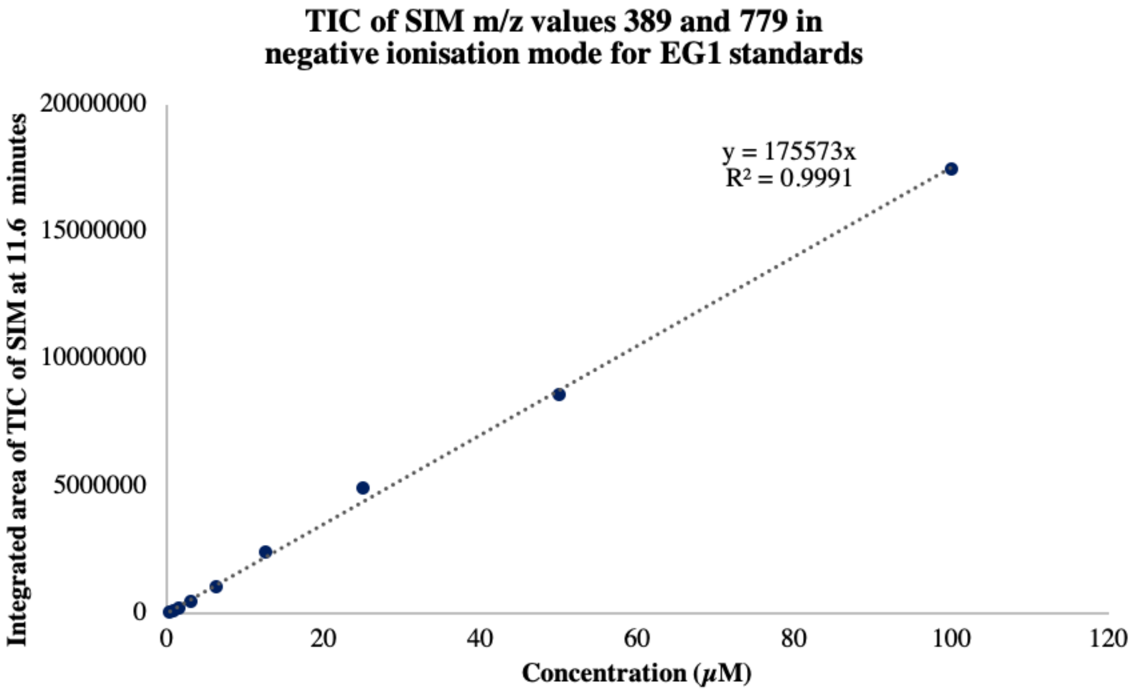

Each data point obtained from the average of 2 injections per standard.

12.3 TIC of SIM *m/z* values 389 and 779 in negative ionisation mode for EG1 standards

2 µL injection of EG1 standards. The label on the chromatogram is the retention time in minutes.

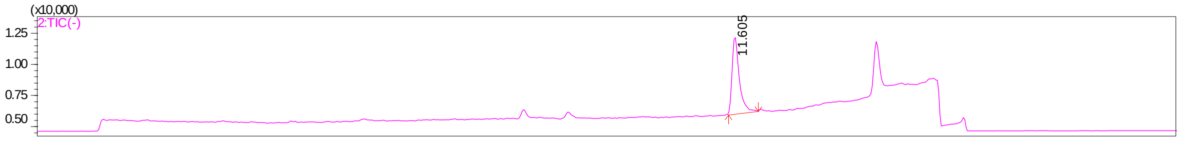

0.39 µM EG1 standard

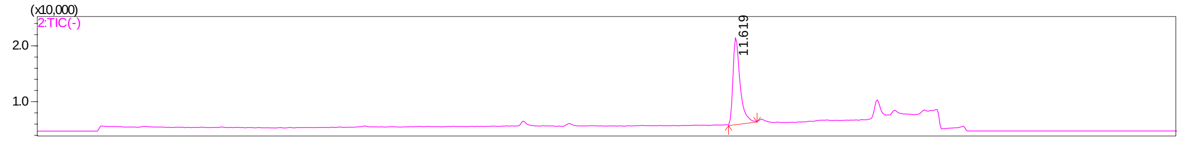

0.78 µM EG1 standard

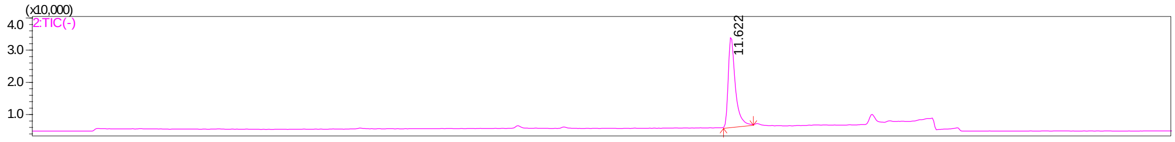

1.56 µM EG1 standard

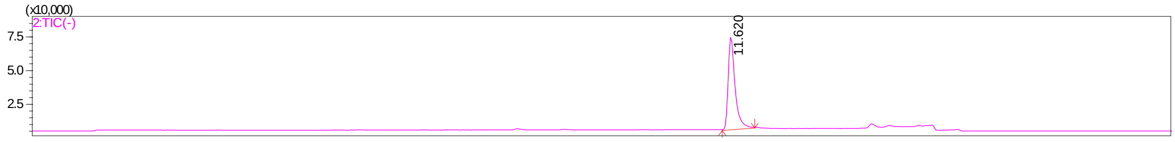

3.13 µM EG1 standard

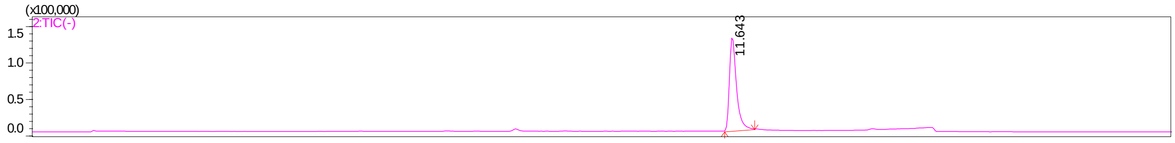

6.25 µM EG1 standard

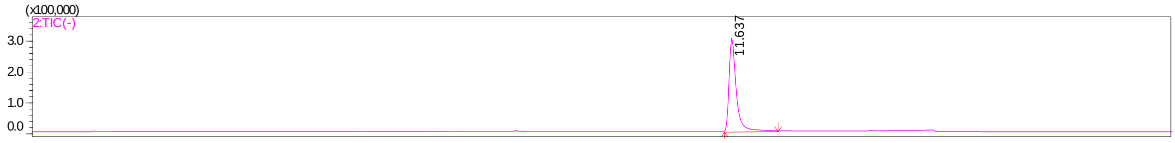

12.5 µM EG1 standard

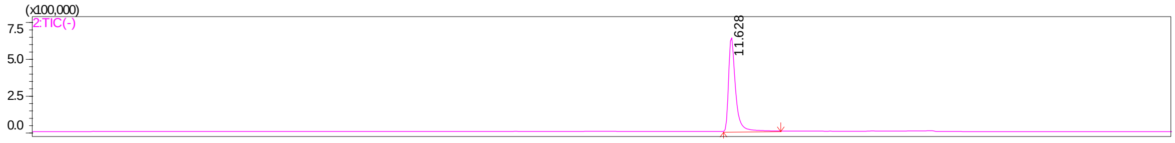

25 µM EG1 standard

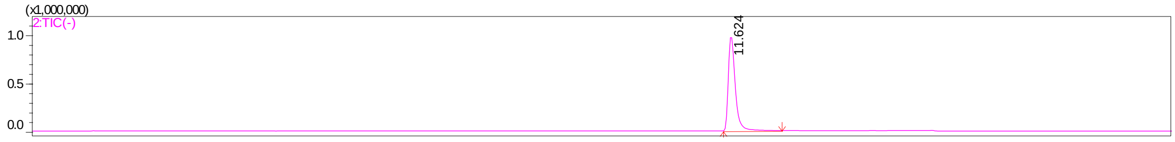

50 µM EG1 standard

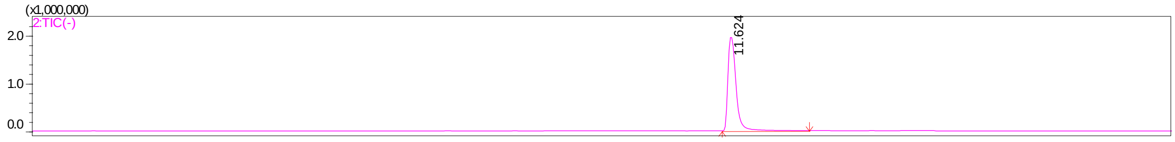

100 µM EG1 standard

12.4 Example of TIC for the SIM *m/z* values 389 and 779 in negative ionisation mode for EG1 lysate and media samples obtained from HCT116 cells treated with 250 µM of EG1.

2 µL injection of samples. The label on the chromatogram is the retention time in minutes.

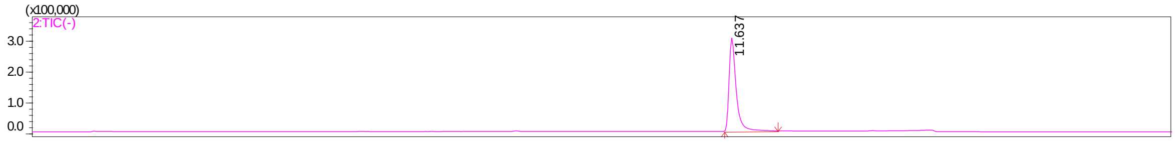

EG1 standard (12.5 µM)

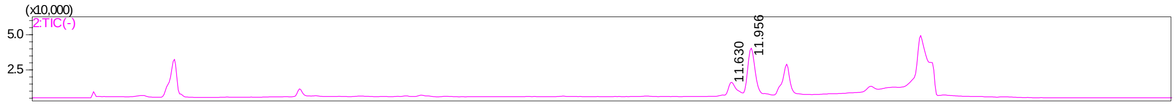

$x = \frac{y}{175573}$ where y = 73944, therefore x = ~0.4 µM

EG1 Lysate

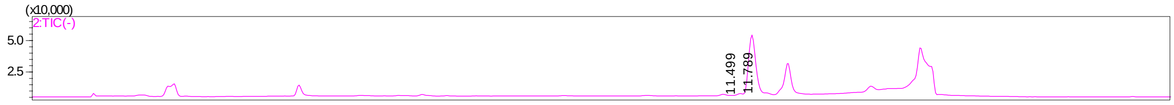

EG1 Lysate control

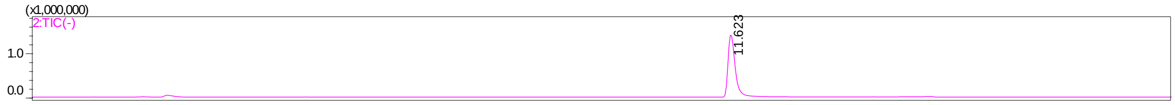

$x = \frac{y}{175573}$ where y = 11612990, therefore x = ~66 µM

EG1 Media

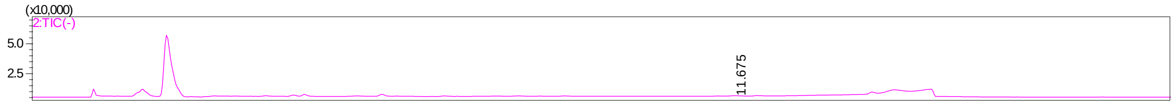

EG1 Media Control

Approximate concentration of EG1 detected (µM)[a]	
EG1 lysate[b]	0.4
EG1 media[b]	66

Approximate concentration of EG1 detected by LC-MS SIM analysis for lysate and media samples obtained from HCT116 cells treated with the high 250µM dosage EG1. [a]Concentrations calculated using equation (2). [b]Lysate and media samples obtained from HCT116 cells treated with 250µM of EG1.

12.5 Example of TIC for the SIM *m/z* values 389 and 779 in negative ionisation mode for EG1 lysate and media samples obtained from HCT116 cells treated with 25 µM of EG1.

2 µL injection of samples. The label on the chromatogram is the retention time in minutes.

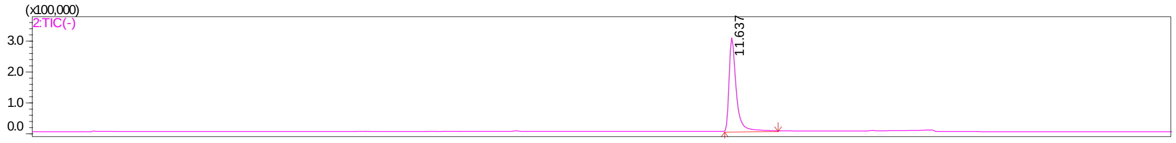

EG1 standard (12.5 µM)

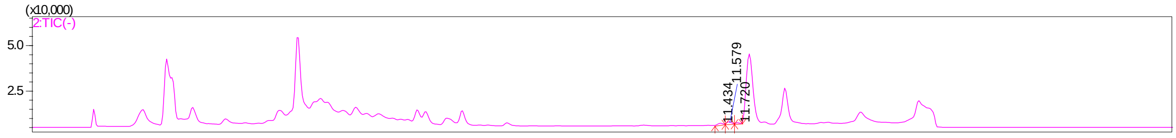

EG1 Lysate

$$x = \frac{y}{175573}$$ where y = 5417, therefore x = ~0.03 µM,

(less than 0.39 µM, the least concentrated standard of EG1).

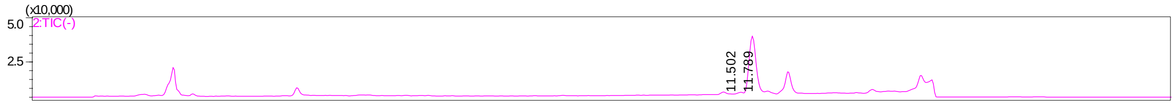

EG1 Lysate Control

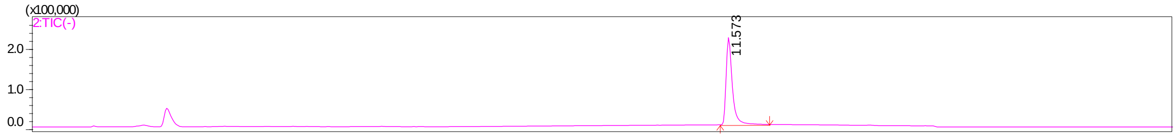

$$x = \frac{y}{175573}$$ where y = 1783113, therefore x = ~10 µM

EG1 post-treatment 24 h media

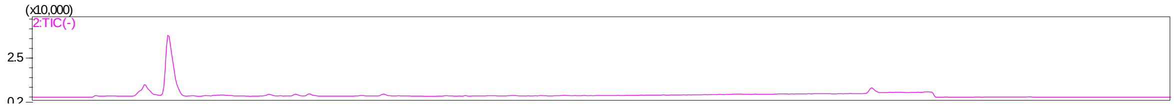

EG1 Control post-treatment 24 h media

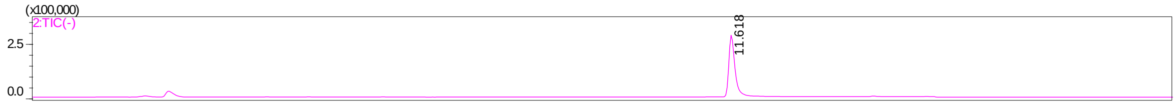

$$x = \frac{y}{175573}$$ where y = 1959035, therefore x = ~11 µM

EG1 pre-treatment media

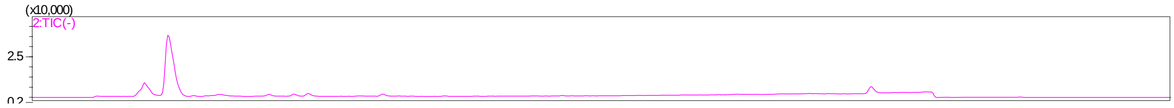

EG1 Control pre-treatment media

12.6 STR profiling reports of the non-transformed HCT116 and the transformed HCT116/FUCCI cell lines

DNA Diagnostics

Cell Line Profiling Report

New Zealand
tel: (09)5710474
fax: (09)5710475
www.dnadiagnostics.co.nz

Report to Niki Hazelton
Niki.Hazelton@otago.ac.nz

Reference	Sample Type	Lab Number	Sample Dated	Sample Received
HCT116	Extracted DNA	20B1711		18/12/20

Reference number : DNA02327_1
Date reported: 23 December 2020

Results:

Cell Line studies were undertaken using the Applied Biosystems AmpFlSTR® Identifiler™ on an Applied Biosystems SeqStudio™ Genetic Analyser

Results:

	D8S1179	D21S11	D7S820	CSF1PO	D3S1358	TH01	D13S317	D16S539	D2S1338	D19S433	vWA
, HCT116	13<br>14	29<br>30	11<br>12	7<br>10	12<br>19	8<br>9	10<br>12	11<br>13	16<br>16	12<br>13	17<br>22

	TPOX	D18S51	D5S818	FGA	Amelogenin
, HCT116	8<br>8	16<br>16	10<br>11	18<br>24	x<br>x

SUMMARY / INTERPRETATION:

Authorised by:

Maria Andres BSc
Registered Medical Laboratory Scientist (30-09596)

The HCT116 cell line was short tandem repeat (STR) profiled to ensure that the culture used for this study has retained the same STR markers as the reference HCT116 cell line. The profiling was performed by DNA diagnostics using the applied biosystems AmpFLSTR identifier or SeqStudio Genetic Analyser to measure STR abundance. The profiling showed that our culture has an 89% similarity to the reference with the only differences occuring in D3S1358, vWA and FGA, therefore we are confident that our cells are of colorectal cancer descent.

DNA Diagnostics

Cell Line Profiling Report

New Zealand
tel: (09)5710474
fax: (09)5710475
www.dnadiagnostics.co.nz

Report to Niki Hazelton
niki.hazelton@otago.c.nz

**Reference**	**Sample Type**	**Lab Number**	**Sample Dated**	**Sample Received**
HCT116-FUCCI	Buccal Cells	20B1712		18/12/20

Reference number : DNA02328_1
Date reported: 23 December 2020

Results:

Cell Line studies were undertaken using the Applied Biosystems AmpFℓSTR® Identifiler™ on an Applied Biosystems SeqStudio™ Genetic Analyser

Results:

	D8S1179	D21S11	D7S820	CSF1PO	D3S1358	TH01	D13S317	D16S539	D2S1338	D19S433	vWA
, HCT116-Fucci	12<br>14	29<br>30	11<br>12	7<br>10	12<br>18,19	8<br>9	10<br>12	11,(12)*<br>13,(14)*	16<br>16	12<br>13	17,(18)*<br>22,(23)*

	TPOX	D18S51	D5S818	FGA	Amelogenin
, HCT116-Fucci	8<br>8	16<br>17,18	10<br>11	18<br>23	x<br>x

SUMMARY / INTERPRETATION:

( ) * = stutter or minor peak

Authorised by:

Andres

Maria Andres BSc
Registered Medical Laboratory Scientist (30-09596)

Identical to our HCT116 cell culture report the HCT116/FUCCI cells have shown an 89% similarity to the reference with differences in D3S1358, vWA and FGA. Therefore, STR profiling of the HCT116/FUCCI cell line has shown that regardless of the construct these cell's have been verified as HCT116's and thus are expected to behave in the same way as the original non-transformed cell line.

12.7 All PAX gene expression in HCT116, UACC62 and PC-3

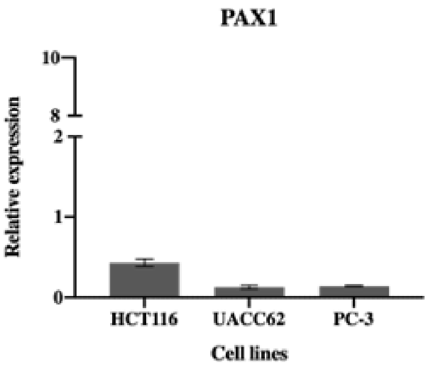

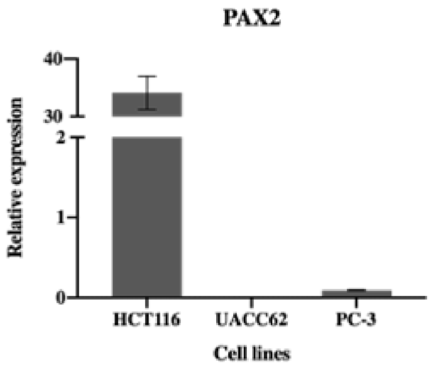

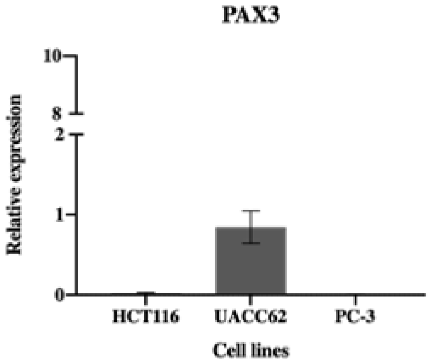

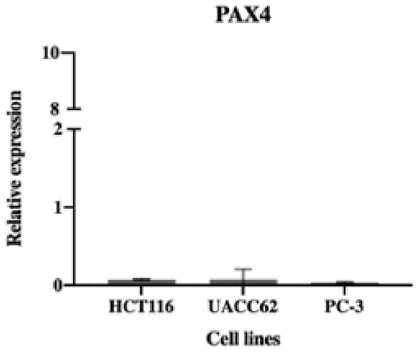

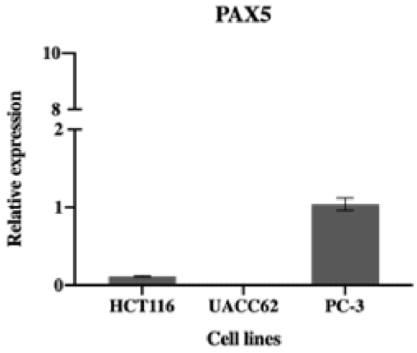

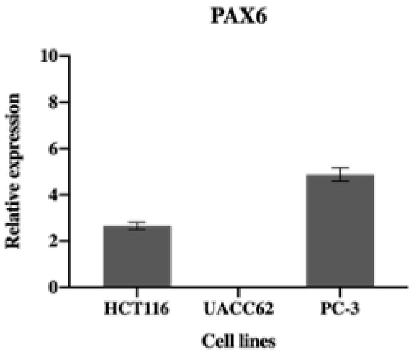

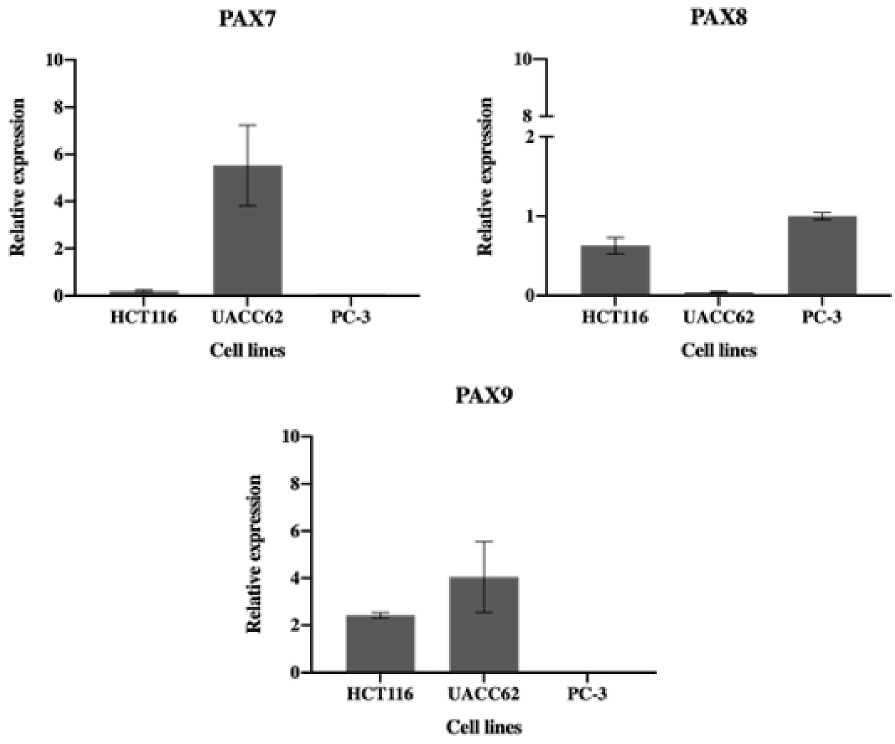

Expression of the nine annotated PAX isoforms in HCT116, UACC62 and PC-3 cell lines have been measured by RT-qPCR to compare HCT116 isoform expression with two other cancer cell lines. We found that the highest PAX expression in HCT116 cells was PAX2, PAX6 and PAX9 overall, however these cells had higher expression of PAX1 and PAX2 than the other cell lines. Additionally, HCT116 has similar levels of PAX8 and PAX9 as PC-3 and UACC62 respectively.

12.8 Table of qPCR primer sequences used in this study

Target	5' primer	3' primer
HPRT1	GAAAGGGTGTTTATTCCTCATGGA	GAAAGGGTGTTTATTCCTCATGGA
B2M	ACTCTCTCTTTCTGGCCTGG	ACGGCAATACTCATCTT
UBC	GCAAAGATCCAAGATAAGGAA	GGACCAAGTGCAGAGTGGAC
PAX1	ACCCCCGCAGTGAATGG	TGTACACGCCGTGCTGGTT
PAX2	CCTGGCCACACCATTGTTC	TCACGTTTCCTCTTCTCACCAT
PAX3	ACGCGGTCTGTGATCGAAACA	TCTCGCTTTCCTCTGCCTCCTT
PAX4	CAGAGGCACTGGAGAAAGAGTTC	CCATTTGGCTCTTCTGTTGGA
PAX5	GTGCCTGGGAGTGAGTTTTCC	GGCGGCAGCGCTATAATAGT
PAX6	GAGGCTCAAATGCGACTTCAG	TGCTAGTCTTTCTCGGGCAAA
PAX7	GGAAGAAAGAGGAGGAGGATGAG	CCAGCCGGTTCCCTTTGT
PAX8	TGAGGGCGTCTGTGACAATG	CGGGACTCAGGGACTTGGT
PAX9	AGTACGGTCAGGCACCAAATG	ATAACCAGAAGGAGCAGCACTGTAG
Δ40P53	TCTGAAAATGTTTCCTGACTC AGA	TTCCCTGGATTGGCAGC
TGF-βR1	ACGGCGTTACAGTGTTTCTG	AACTTTGTCTGTGGTCTCTGTGA
CDH2	TGAGGAGTCAGTGAAGGAGTCA	GCAAGTTGATTGGAGGGATG

12.9 Flow cytometry single stain data

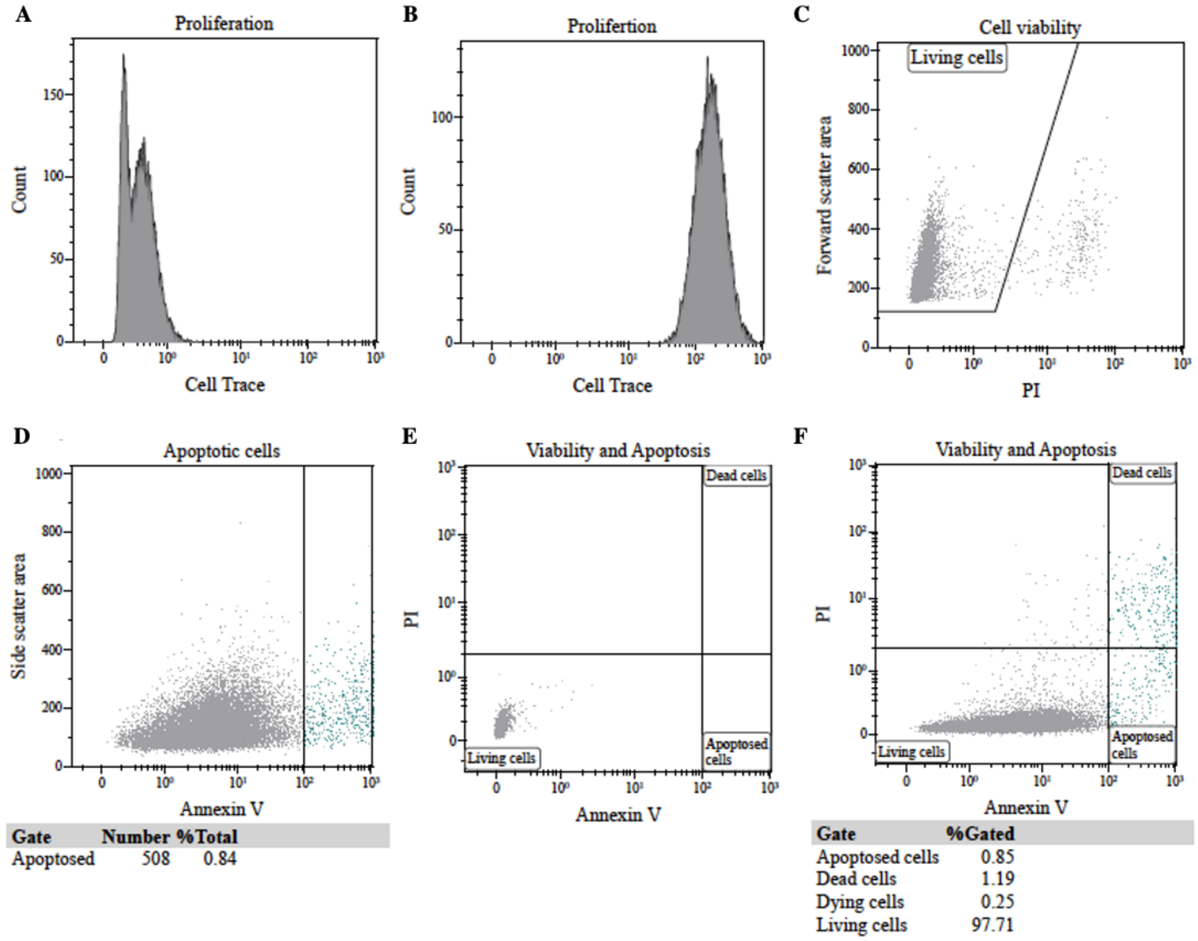

An example of single stain flow cytometry data used to adjust the voltages and set the viability and apoptosis gates for the control and EG1 treated samples. **A)** Cell trace violet in the unstained cells shows that without staining the cells are unable to be detected and so will reliable produce results in the treatment populations. **B)** A full stain cell trace violet control was used to identify the upper limit of staining in cells which have not yet divided. **C)** Propidium iodide staining of a mixed population of living and heat treated dead cells allowed us to gate on the viable cells to ensure that only these cells were used in the proliferation plot. **D)** Positive Annexin V 647 staining of an apoptosis induced population was used to determine the fluorescence intensity of apoptosing cells thus measuring the extent of apoptosis with EG1 treatment. **E)** Propidium iodide versus Annexin V in the unstained population was unable to identify cells in the dead or apoptotic quadrants when the gates were determined by the single stained plots. **F)** Staining of both Propidium iodide and Annexin V produced cell populations in the three main quadrants; dead cells, living cells and apoptosed cells.

12.10 Flow cytometry proliferation calculation and analysis

FACS proliferation plots

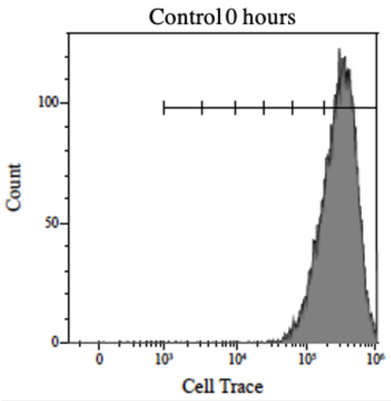

Gate	Number	%Gated
0	12,427	81.24
1	2,717	17.76
2	124	0.81
3	7	0.05
4	4	0.03
5	8	0.05

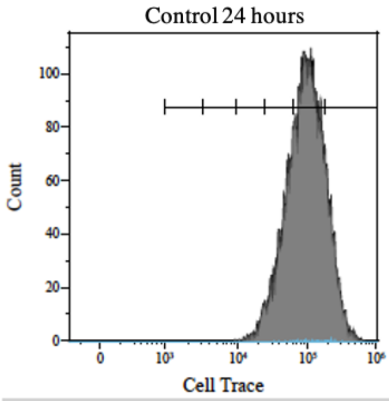

Gate	Number	%Gated
0	2,634	16.41
1	9,317	58.06
2	3,805	23.71
3	322	2.01
4	2	0.01
5	0	0.00

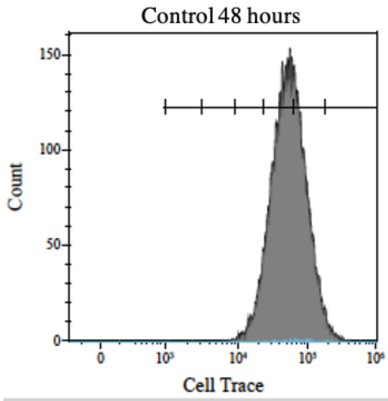

Gate	Number	%Gated
0	352	1.77
1	7,493	37.66
2	10,762	54.08
3	1,385	6.96
4	17	0.09
5	3	0.02

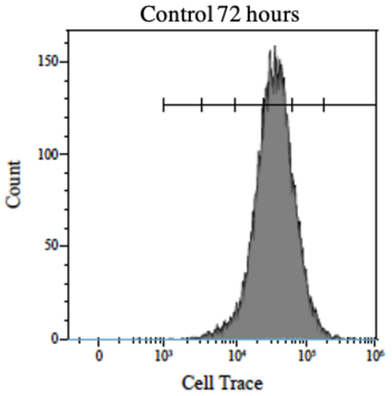

Gate	Number	%Gated
0	124	0.57
1	3,432	15.77
2	12,878	59.19
3	5,018	23.06
4	591	2.72
5	26	0.12

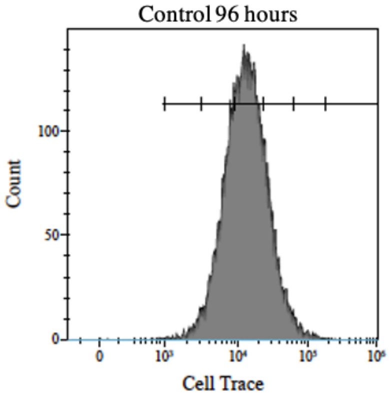

Gate	Number	%Gated
0	9	0.04
1	322	1.41
2	4,111	18.06
3	11,815	51.91
4	6,598	28.99
5	413	1.81

Raw values

	Control				EG1 treated			
**Divisions**	**24h**	**48h**	**72h**	**96h**	**24h**	**48h**	**72h**	**96h**
**0**	4940	196	9	7	7476	82	32	48
**1**	11180	4469	285	108	11796	4575	1070	713
**2**	3415	6937	5495	1813	2174	12648	9262	6629
**3**	88	2049	10621	9685	34	4914	9817	10033
**4**	1	221	4591	8305	0	59	1809	2769
**5**	0	4	81	431	0	2	7	7
**Total**	19624	13871	21001	19918	21479	22277	21991	20192

Adjusted values (precursor cohort model)

	Control				EG1 treated			
**Divisions**	**24h**	**48h**	**72h**	**96h**	**24h**	**48h**	**72h**	**96h**
**0.5**	4940	196	9	7	7476	82	32	48
**1.5**	5590	2234	143	54	5898	2288	535	356
**2.5**	854	1734	1374	453	543	3162	2316	1657
**3.5**	11	256	1328	1211	4	614	1227	1254
**4.5**	0	14	287	519	0	4	113	173
**5.5**	0	0	3	13	0	0	0	0

Proliferation plots were produced at each time point for both the control and EG1 treated HCT116 cells to compare the shift in CellTrace over time and therefore the difference in cell division with EG1 treatment. The control cell population was used to set the gates in which defined each division by using the peak median at each time point as the gate boundaries. Kaluza analysis (version 2.1.1, 2018) was then used to measure the number of cells that were included within each gate across the four time points. These values were used to calculate the proportion of each population between the five divisions identified by the gates to produce the raw values. The raw values were then used in the precursor cohort model equation decribed by Hawkins et al. (2007) which gives identifies a distribution in the data that takes into account the likelihood that the cells are between divisions (see section 2.8). The adjusted values were plotted using Rstudio (version 1.4, 2020) for a final comparison of proliferation in the EG1 treated and control cell populations.

12.11 Preliminary PC-3 proliferation and apoptosis data shows minimal change between the control and EG1 treated cultures.

Proliferation

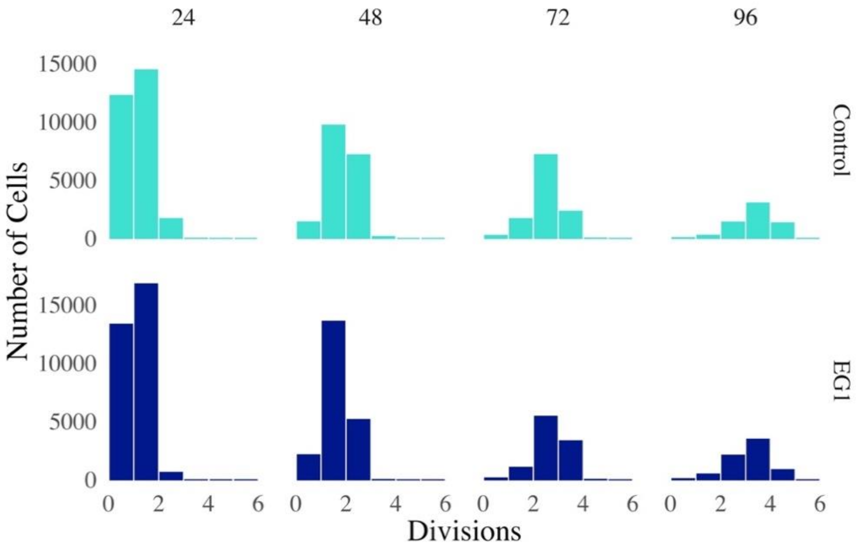

Apoptosis

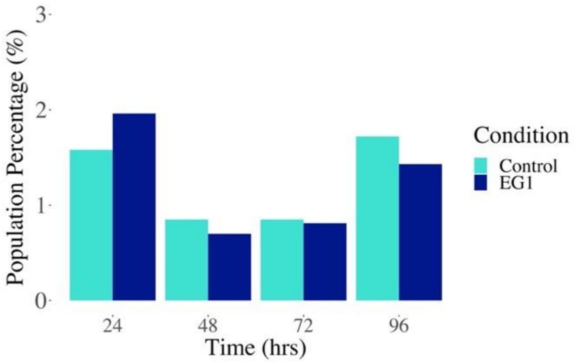

EG1 has previously been described as primarily being a PAX2 inhibitor (Grimley et al. 2017) and so to determine if this compound preferentially inhibits PAX2 over other structurally similar PAX proteins we measured proliferation and apoptosis in the PC-3 cell line. PC-3 cells were used as an indicator of this as they express low levels of PAX2 and higher levels of PAX6 and PAX8 compared to the HCT116's. Thus, analysis of proliferation in these cells has shown that at 48 hours there were 47% more control cells than EG1 treated that had undergone two divisions, however at 72 hours 30% more EG1 treated cells than controls had progressed through three divisions. Finally, at 96 hours there was minimal difference between the control and EG1 treated populations, suggesting that EG1 has more impact on PAX2 function over other PAX proteins. Additionally, there was minimal apoptosis in the EG1 treated or control cell populations, with 1.96% in the EG1 treated population at 24 hours being the highest rate overall, and the largest difference being 0.38% more apoptosis in the EG1 treated cells after 24 hours when compared to the controls.

12.12 Cell cycling flow cytometry visualization of data and analysis

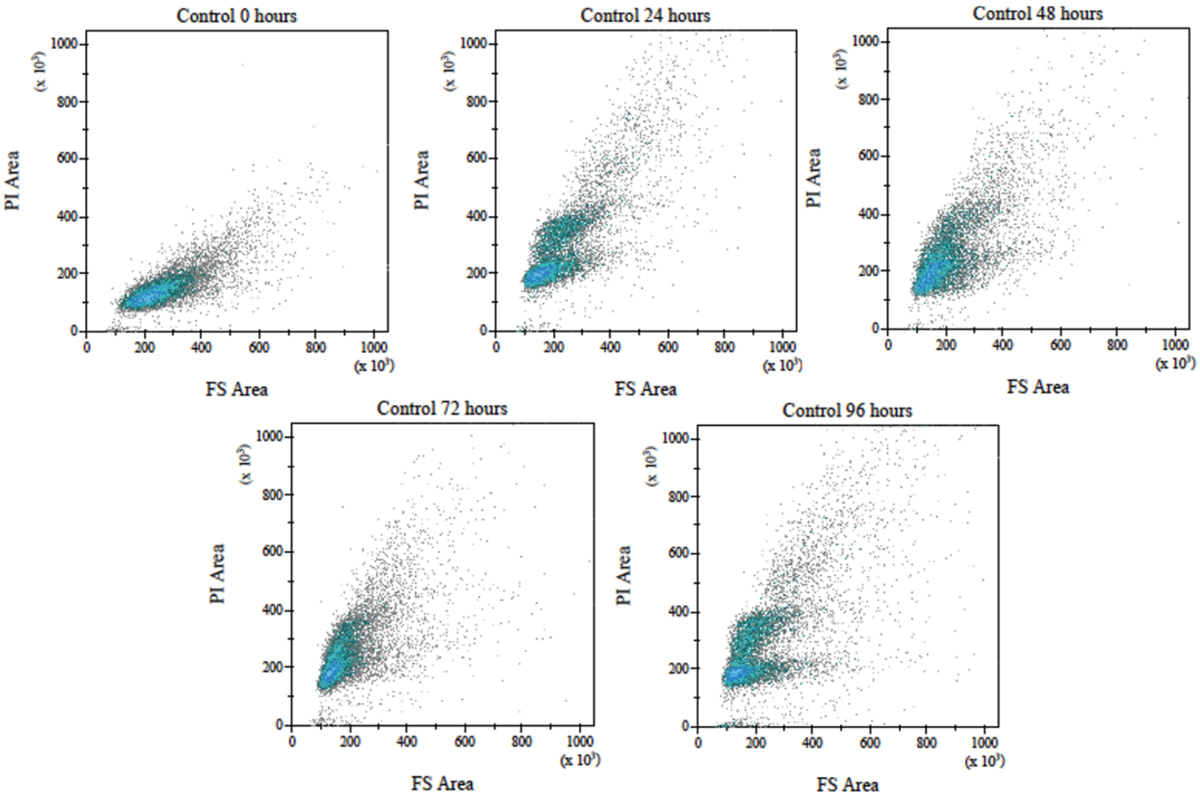

The scatter plots produced by propidium iodide (PI) versus forward scatter area for each population were used to identify the distribution of cells within and between the cell cycle phases. By using cell density this plot gave a better representation of population shift than the histogram and was able to show that by using PI we could identify the movement of HCT116's through the cell cycle.

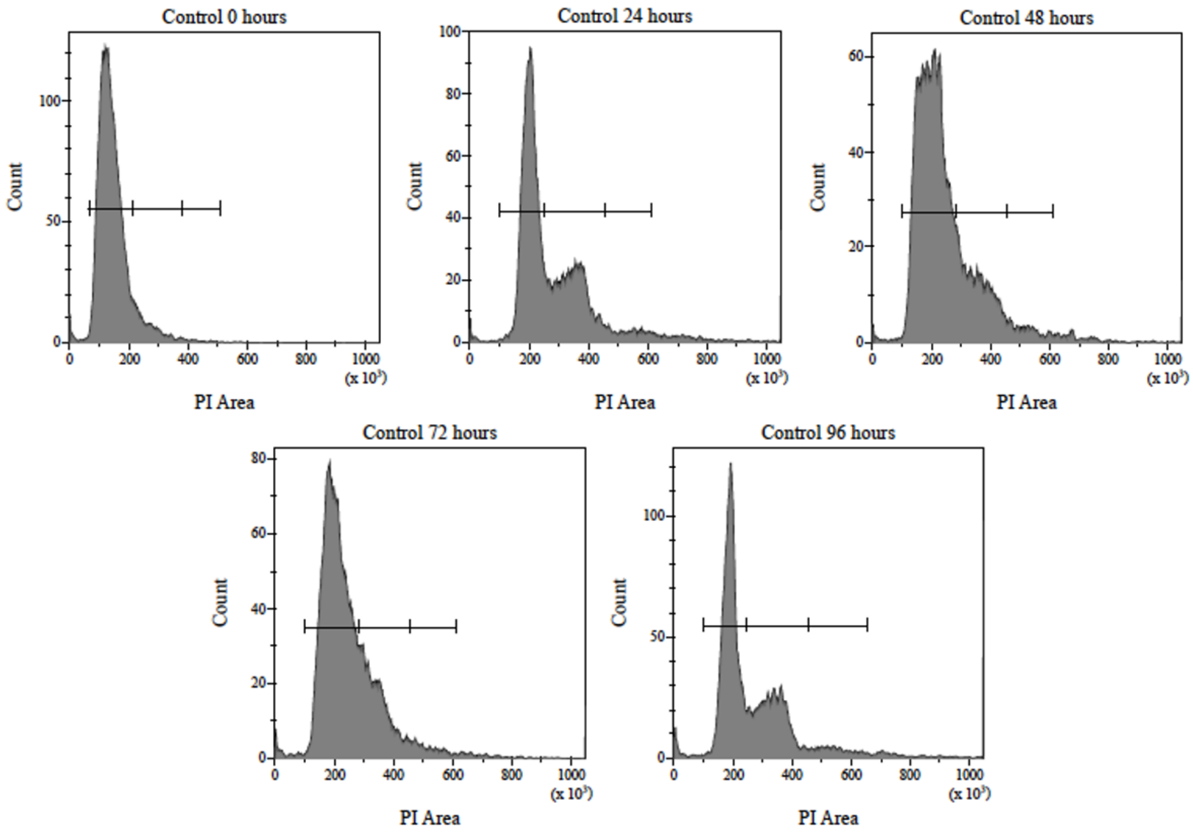

Histograms of cell count versus PI were produced during cell collection to identify changes in population percentages per phase over 96 hours of treatment in the EG1 treated compared to the controls. The percentage of cells in each phase was determined by the gates set using the full stain control for each time point by identifying the fluorescent peak boundaries which represent the G0/G1, S and G2/M phases. The percentage of cells which lie within the gates for each phase was then used to determine a change in cell cycling with EG1 treatment.

Supplementary material references

1. Hamuro, Y. H.; Geib, S. J.; Hamilton, A D. *J. Am. Chem. Soc*. **1996**, *118*, 7529-7541.
2. Prashanth, M. K; Revanasiddappa, H. D. *Med. Chem. Res.* **2013**, *22*, 2665-2676.
